# Various forms of double burden of malnutrition problems exist in rural Kenya

**DOI:** 10.1186/s12889-019-7882-y

**Published:** 2019-11-21

**Authors:** Andrea Fongar, Theda Gödecke, Matin Qaim

**Affiliations:** Department of Agricultural Economics and Rural Development, University of Goettingen, 37073 Goettingen, Germany

**Keywords:** Double burden of malnutrition, Undernutrition, Overweight, Micronutrient deficiency, Rural Kenya

## Abstract

**Background:**

The coexistence of overweight/obesity and undernutrition is often referred to as the double burden of malnutrition (DB). DB was shown to exist in many developing countries, especially in urban areas. Much less is known about DB in rural areas of developing countries. Also, the exact definition of DB varies between studies, making comparison difficult. The objective of this study is to analyse DB problems in rural Kenya, using and comparing different DB definitions and measurement approaches.

**Methods:**

Food intake and anthropometric data were collected from 874 male and female adults and 184 children (< 5 years) through a cross-section survey in rural areas of Western Kenya. DB at the individual level is defined as a person suffering simultaneously from overweight/obesity and micronutrient deficiency or stunting. DB at the household level is defined as an overweight/obese adult and an undernourished child living in the same household, using underweight, stunting, wasting, and micronutrient deficiency as indicators of child undernutrition.

**Results:**

DB at the individual level is found in 19% of the adults, but only in 1% of the children. DB at the household level is relatively low (1–3%) when using wasting or underweight as indicators of child undernutrition, but much higher (13–17%) when using stunting or micronutrient deficiency as indicators.

**Conclusion:**

Various forms of DB problems exist in rural Kenya at household and individual levels. Prevalence rates depend on how exactly DB is defined and measured. The rise of overweight and obesity, even in rural areas, and their coexistence with different forms of undernutrition are challenges for food and nutrition policies.

## Background

Various nutritional problems exist in developing countries. More than 800 million people are still affected by chronic undernutrition, at least 1.5 billion people suffer from micronutrient deficiencies [1, 2]. At the same time, rates of overweight and obesity are increasing rapidly in many places [1–3]. The coexistence of undernutrition and overweight/obesity in the same countries, communities, and households is known as the double burden of malnutrition (DB) [4]. Recent research has analysed the DB phenomenon in different geographical regions [2, 5–11]. Many existing studies analyse DB within households by looking at pairs of overweight mothers and undernourished children. Reported prevalence rates of this type of DB problem within households range from less than 5% in sub-Saharan Africa to 30% in Malaysia [12, 13]. In specific locations and population groups, the prevalence of DB households can be higher. A recent study found a DB rate of 43% among poor households in Nairobi, Kenya [14]. Hardly any specific data on the DB phenomenon are available for rural areas, where the situation may be different from urban areas. Better understanding malnutrition and DB problems in rural areas is important for planning rural food and nutrition policies. This is especially true in Africa, where many of the poor and undernourished people still reside in rural areas.

One important question is what concrete indicators to use when analysing DB phenomena. No uniform definition of DB indicators exists. In most studies of DB households, a mother is commonly classified as overweight when her body mass index (BMI) exceeds 25.0 kg/m^2^ [15]. Yet, the classification of undernourished children differs between studies in terms of the indicators used and the cut-off points applied [8]. Indicators of child undernutrition used in existing DB research are weight-for-age Z-scores (WAZ) [13, 16], height-for-age Z-scores (HAZ) [14, 17–20], BMI-for-age Z-score (BAZ) [6], simple BMI [5], or combinations of WAZ, HAZ and weight-for-height Z-scores (WHZ) [21, 22]. Given these differences in measurement, comparability of studies is difficult. It remains unclear whether differences in the reported DB rates across countries are real differences or mainly driven by different definitions and measurement approaches. Against this background, comparing different definitions in the same setting can be a useful exercise.

Anthropometric indicators are suitable to establish undernutrition in a calorie sense, but less to identify micronutrient deficiency. Micronutrient deficiencies are better analysed with blood samples or dietary data. Broadening the perspective to include micronutrients, DB households can also be households in which one member is overweight or obese while another member is micronutrient-deficient. In addition, the DB phenomenon can be observed within individuals that suffer simultaneously from overweight/obesity and micronutrient deficiency. The literature on individual-level DB problems is relatively thin. A few studies have analysed the coexistence of overweight and iron or zinc deficiency in children, adolescents, and women of reproductive age in selected countries and regions [20, 23–26]. For rural Africa, related research hardly exists. Children can also suffer simultaneously from overweight/obesity and stunting, which is yet another individual-level DB definition. This co-occurrence of overweight/obesity and stunting is sometimes also called the ‘short- and plump syndrome’ [2, 10, 19, 23]. Over 8 million children under the age of 5 years suffer from this syndrome globally, although most of them live in regions other than Africa [2].

This article contributes to the research direction by analysing DB problems in rural areas of Kenya. As mentioned, research on DB phenomena in rural areas of developing countries is rare. Official statistics for Kenya show that overweight and undernutrition coexist at national level [27], but no specific DB data for households in rural Kenya exist. The main objective of this article is to determine the prevalence of DB problems in rural Kenya by using and comparing different definitions and measurement approaches. First, we examine individual-level DB in male and female adults and children by looking at overweight and micronutrient deficiency or stunting. Second, we explore household-level DB by looking at adult-child pairs, using different measures of child undernutrition, including underweight, stunting, wasting, and micronutrient deficiency.

## Methods

### Study context and data

The data for this research were collected through a survey of rural households in the Nyanza region in Western Kenya. In spite of nutritional improvements during the last 20 years, the prevalence of child undernutrition remains high in rural Kenya [27, 28]. According to official statistics, around 25% of the children < 5 years were stunted in the Nyanza region in 2015. At the same time, almost 30% of the female adults were classified as overweight or obese [27].

The survey for this research was administered in 2016 in rural areas of Kisii and Nyamira Counties, where nearly all households are involved in subsistence-oriented farming [29]. Power calculations showed that we should target at least 750 households to analyse expected prevalence rates of malnutrition at a confidence level of 95% and a margin of error of 3%. As no recent household census was available, we used a two-stage random sampling procedure to obtain representative data. Most households in the study region are organized in farmer or self-help groups. We obtained a list of all 107 groups registered with the Ministry of Gender, Children, and Social Development in the study region. Out of this list, we randomly selected 48 groups. The groups varied in size, but on average one group had about 25 member households. We used complete membership lists to randomly select 15–20 member households in each group, depending on group size. Thus, we obtained a total sample of 835 households, which exceeds the required number of target households from the power calculations. The household sample is representative of rural areas in Kisii and Nyamira.

We interviewed household heads in the local language using a structured questionnaire that was specifically developed for this project (see Additional file 1). In addition to questions related to nutrition and food consumption, the questionnaire also captured data on general household characteristics (household members, economic activities etc.). Specially recruited and trained enumerators from the region carried out the interviews. In addition to the household head, a second adult of the opposite sex, and one child aged 6–59 months were selected to collect individual-level food intake data and anthropometric measures. The second adult targeted was the household head’s spouse. When no spouse existed, another opposite-sex adult in the household was randomly selected, whenever available. When more than one child in the target age group was living in the household, the subject child was also selected randomly. Many of the households did not have young children and/or only one adult (> 16 years). Nutritional data were obtained for 1058 individuals (558 female adults, 316 male adults, 184 children). Data on adult-child pairs were available for 173 households. Data from pregnant women were excluded from the analysis. Written informed consent was obtained from household heads and the parents of participating children. We followed recommended international ethical guidelines.

The small number of young children living in the target households was unanticipated and is due to the relatively old mean age of the household heads and their spouses (see below). Of course, the smaller child and adult-child pair subsamples reduce the reliability of the estimates. However, ex post power calculations showed that a sample size of 180 is still sufficient to estimate malnutrition prevalence rates with a margin of error or 7% (confidence level of 95%). Hence, while the exact point estimates should not be over-interpreted, a reasonable estimate of the order of magnitude is still possible also with these smaller subsamples.

### Measuring nutritional status

Weight and height/length measures were taken from all individuals, following recommended techniques [30, 31] and under supervision of the researchers. For children younger than 24 months, length was taken lying using a portable infantometer (seca 417 Height Measuring Board). Standing height was taken using the seca stadiometer 217. Weight was measured using a normal weighing scale. For small children (< 24 months), the weight of the caregiver with and without carrying the child was taken and the difference was calculated. All measures were taken twice; the mean of both measures was used for the computation of nutritional status indicators.

For adults, we calculated the BMI. Cut-off points for nutritional status categories are applied as recommended by the World Health Organization (WHO) [32]; < 18.5 kg/m^2^ for underweight, 25.0–29.9 kg/m^2^ for overweight, and ≥ 30.0 kg/m^2^ for obese. For children, height-for-age Z-scores (HAZ), weight-for-height Z-scores (WHZ), weight-for-age Z-scores (WAZ), and body-mass-index-for-age Z-scores (BAZ) were calculated, using the 2006 WHO growth standard reference [33]. Stunting, wasting, and underweight are respectively defined as HAZ, WHZ, and WAZ being below the cut-off of − 2 standard deviations (SD) [33, 34]. As an alternative measure of child underweight, BAZ < − 2 SD was also used. Child overweight was defined as WHZ > + 2 SD, and alternatively as BAZ > + 2 SD [33–35]. In addition, being at risk of overweight (BAZ > + 1 SD, WHZ > + 1 SD) was calculated. Implausibly high and low data (22 data sets) were flagged and excluded, using the proposed cut-off points of −/+ 6 SD for WAZ and −/+ 5 SD for WHZ and HAZ [33, 35].

### Measuring micronutrient deficiency

Micronutrient deficiency was analysed with individual-level food intake data. The focus was on vitamin A, iron, and zinc, as these three micronutrients make up the largest share of the health problems caused by micronutrient deficiencies in developing countries [36, 37]. Iodine deficiency is also relevant but less straightforward to determine with dietary recall data, because iodine fortification of salt is common, and survey respondents typically find it difficult to state the exact quantity of salt consumed. In Kenya, iodine fortification of salt is mandatory [38]. Individual-level food intake was captured using 24-h dietary recalls. These recalls were collected twice from the adults on two non-consecutive days. Special days (e.g., celebrations) were excluded. For children, a single dietary recall was answered by the caregiver. Food quantities eaten were converted into micronutrients using the Kenyan and Tanzanian food composition tables [39, 40]. For a few food items not listed in these tables, other food composition tables were used [41–43].

To identify micronutrient deficiency, estimated average requirements (EAR) were used for each nutrient, taking individual gender and age into account [44]. An individual was defined as micronutrient-deficient if the intake of at least one of the three micronutrients was below the individual EAR. Additional file 2: Table S2 shows the EAR thresholds used to define micronutrient deficiency.

### Double burden definitions

Eight different DB definitions were defined and compared (Table 1). The first three definitions (DB 1, DB 2 and DB 3) refer to the individual level, using all available data from adults and children in the sample. Individual-level DB means that a person suffered simultaneously from overweight/obesity and micronutrient deficiency. In the definition DB 3, we look at the coexistence of overweight/obesity and stunting in children. The other DB definitions (DB 4 to DB 8) refer to the household level, focusing on adult-child pairs. A household is defined as a DB household if one adult is overweight or obese and one child is undernourished. The household-level definitions vary in terms of the child undernutrition indicators, namely underweight (based on WAZ and BAZ), stunting, wasting, and micronutrient deficiency (Table 1).

**Table 1:** Definitions used to define double burden of malnutrition (DB) at individual and household levels

DB	Definition
Individual level ^a^	
DB 1	=1 if adult overweight/obese (BMI ≥25.0 kg/m^2^) and micronutrient-deficient
DB 2	=1, if child overweight (BAZ > + 2 SD) and micronutrient-deficient
DB 2.1	=1, if child overweight (WHZ > + 2 SD) and micronutrient-deficient
DB 3	=1, if child overweight (BAZ > + 2 SD) and stunted (HAZ < -2 SD)
DB 3.1	=1, if child overweight (WHZ > + 2 SD) and stunted (HAZ < -2 SD)
Household level ^b^	
DB 4	=1, if adult overweight/obese (BMI ≥25.0 kg/m^2^) and child underweight (BAZ < -2 SD)
DB 5	=1, if adult overweight/obese (BMI ≥25.0 kg/m^2^) and child underweight (WAZ < -2 SD)
DB 6	=1, if adult overweight/obese (BMI ≥25.0 kg/m^2^) and child stunted (HAZ < -2 SD)
DB 7	=1, if adult overweight/obese (BMI ≥25.0 kg/m^2^) and child wasted (WHZ < -2 SD)
DB 8	=1, if adult overweight/obese (BMI ≥25.0 kg/m^2^) and child micronutrient-deficient

^a^ Individual sample: adults *n* = 874 (558 females, 316 males), children *n* = 184

^b^ Household sample (*n* = 173) refers to households with at least one adult (male or female) and one child

The main objective of the analysis is to analyse the prevalence of different types of DB problems. However, in addition it is also interesting to understand what types of individuals and households are particularly affected. This is examined by comparing selected sociodemographic characteristics of individuals and households with and without DB and using simple *t*-tests to identify significant mean differences. This simple comparison cannot identify drivers of DB, but it points at correlations between DB problems and sociodemographic characteristics.

The household-level DB analysis was based on the 173 households with data available for at least one adult (male or female) and one child. In some of the households (*n* = 72), data for two adults (male and female) were available. In a robustness check, these households with data from two adults were looked at separately to see whether the DB prevalence rates change considerably. In a further robustness check, female adult and child pairs (*n* = 164) were analysed separately, to provide a better comparison to previous studies that often focused on mother-child pairs [16–23]. The analysis was performed with the statistical software package Stata version 15.1.

## Results

### Sociodemographic characteristics

Table 2 displays sociodemographic characteristics of the sample. The main economic activity of households in the study region is farming. Farms are very small with an average farm size of 1.3 acres. Around 20% of the households fall below the international poverty line of 1.90 dollar per capita and day. Adults in the sample have an average age of 46.2 years, whereas children have an average age of 35.5 months.

**Table 2:** Sociodemographic characteristics of adults and children in the sample

	Adults (*n* = 874)	Children (*n* = 184)
Individual age (years/months) ^a^	46.2 (12.9)	35.5 (12.7)
Individual sex (male = 1)	0.4 (0.5)	0.5 (0.5)
Kisii county (1/0)	0.7 (0.5)	0.7 (0.5)
Household size (count)	5.1 (1.9)	5.9 (1.7)
Years of formal education	8.7 (3.5)	
Income per capita in PPP $/year	4164.2 (12,911.8)	3469.0 (3863.3)
Poverty rate (1/0)	0.2 (0.4)	0.2 (0.4)
Farm size (acres)	1.3 (1.2)	1.3 (1.2)

Mean values are shown with standard deviations in parentheses

^a^ Age in years for adults and in months for children

### Individual nutrition status

**Fig. 1:**
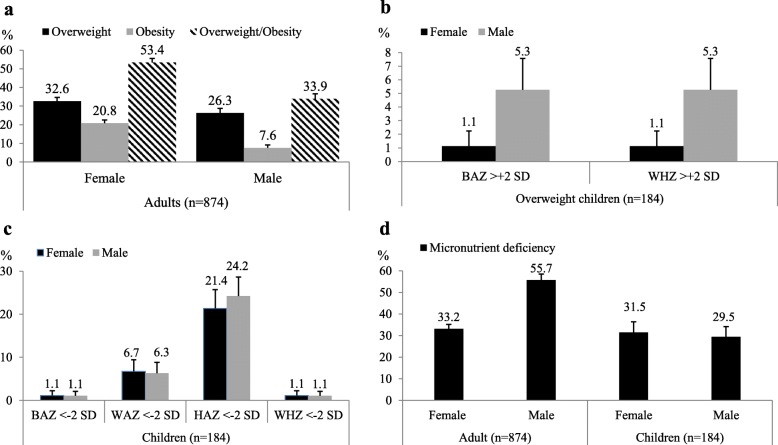
Individual nutritional status. Prevalence of overweight and obesity among adults (**a**). Prevalence of overweight among children (**b**). Prevalence of child underweight, stunting, and wasting (**c**). Prevalence of micronutrient deficiencies among adults and children (**d**). Error bars shown are standard errors

Mean BMI of adults in the sample is 25.2 kg/m^2^, with 46% being either overweight or obese. Figure 1a shows the prevalence of overweight and obesity separately for male and female adults. More than half of the female adults are either overweight or obese. For male adults, the prevalence of overweight and obesity is somewhat lower at about 34%. In spite of these high overweight/obesity rates, about 5% of the adults are classified as underweight (Additional file 3: Table S3), underlining the coexistence of different nutritional problems in the same geographical setting of rural Africa.

For children, depending on the indicator used, 22–28% are classified as at risk of overweight, and 3% as overweight (Fig 1b). At the same time, around 6% of the children are underweight and 23% are stunted (Fig 1c). Around 9% of the children are severely stunted with HAZ < -3 SD (Additional file 3: Table S3). Rates of child wasting are relatively low, about 1% for both boys and girls.

In terms of micronutrient deficiencies, about 40% of the adults have insufficient intakes of vitamin A, iron, or zinc. This is mainly driven by zinc deficiency, which is more widespread in the study region than vitamin A and iron deficiencies (Additional file 4: Table S4). Interestingly, male adults are more affected by micronutrient deficiency than female adults (Fig. 1d). For the child sample, around 30% are classified as micronutrient-deficient, with similar rates for boys and girls. Again, this is largely driven by zinc deficiency.

### Individual-level double burden

**Fig. 2:**
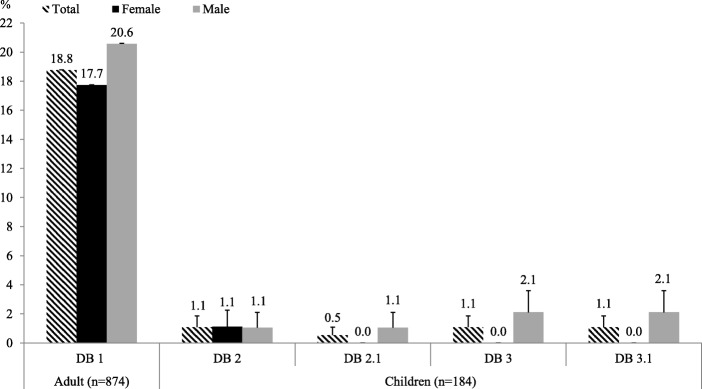
Prevalence of individual-level double burden (DB) in adults and children. Sample size of female adults (*n* = 558). Sample size of male adults (*n* = 316). Sample size of female children (*n* = 89). Sample size of male children (*n* = 95). DB 1, adult is overweight/obese (BMI ≥25.0 kg/m^2^) and micronutrient-deficient; DB 2, child is overweight (BAZ > + 2 SD) and micronutrient-deficient; DB 2.1, child is overweight (WHZ > + 2 SD) and micronutrient-deficient. DB 3, child is overweight (BAZ > + 2 SD) and stunted; DB 3.1, child is overweight (WHZ > + 2 SD) and stunted. Error bars shown are standard errors

Figure 2 shows that 19% of the adults in the sample are affected by individual-level DB (DB 1), meaning that they suffer simultaneously from overweight/obesity and micronutrient deficiency. The rate is slightly higher for male adults (21%) than for female adults (18%). For children, individual-level DB is much lower at 1% when child overweight is measured with BAZ (DB 2) or WHZ (DB 2.1). Stunting and overweight (DB 3 and DB 3.1) also occur in 1% of the children simultaneously. Boys are more affected by individual-level DB than girls.

Additional file 5: Table S5 and Additional file 6: Table S6 compare key sociodemographic characteristics of individuals with and without DB problems. Older adults are more affected by individual-level DB than younger adults (Additional file 5: Table S5). Significant differences are also observed in terms of location; adults in Kisii County are more likely to be affected by DB than adults in Nyamira County. However, there is no significant difference in terms of household income or education, suggesting that there is no clear relationship between adult DB problems and socioeconomic status. For children, almost all of the differences are not statistically significant because the DB prevalence rates among children are very low (Additional file 6: Table S6).

### Household-level double burden

**Fig. 3:**
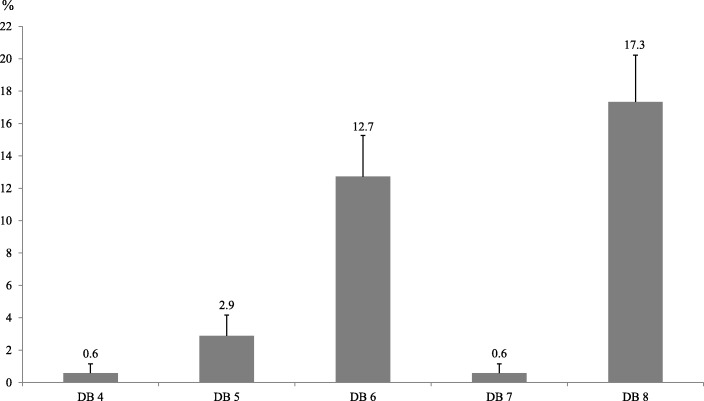
Prevalence of household-level double burden (DB). All double burden definitions (DB 4–8) include adult overweight/obesity (BMI ≥25.0) and child undernutrition, but differ in terms of the child undernutrition indicators used. DB 4, child underweight (BAZ < -2 SD); DB 5, child underweight (WAZ < -2 SD); DB 6, child stunting (HAZ < -2 SD); DB 7, child wasting (WHZ < -2 SD); DB 8, child is micronutrient-deficient. Error bars shown are standard errors

Household-level DB problems exist in rural Kenya, regardless of the concrete DB definition and measurement approach used (Fig. 3). However, the prevalence rates differ remarkably by DB definition. The rates are relatively low (less than 3%) when child undernutrition is measured in terms of underweight (DB 4 and DB 5) and wasting (DB 7), which is due to the fact that relatively few children in the study area suffer from underweight and wasting. Household-level DB rates are much higher (13–17%) when child undernutrition is measured in terms of stunting (DB 6) or micronutrient deficiency (DB 8). These patterns remain similar when restricting the sample to households with two adults and a child, and when only focusing on female adult and child pairs (Additional file 7: Table S7).

Additional file 8: Table S8 compares key characteristics of households with and without DB problems. A significant difference in terms of household income is only observed for DB 8. However, some differences are observed in terms of educational levels: households with DB problems tend to have lower educational levels than households without DB problems.

## Discussion

Multiple forms of malnutrition exist. What contributes to the complexity is the fact that undernutrition, micronutrient deficiency, and overweight/obesity coexist in the same settings, often in the same households and sometimes even in the same individuals. Little is known about double burden (DB) issues in rural Africa. This study has analysed the prevalence of DB in rural areas of Kenya, using and comparing different DB definitions and measurement approaches. Results indicate that DB problems exist in rural Kenya, even though the magnitude varies depending on the concrete definition used.

Individual-level DB was observed in 19% of the adults. The relatively high DB prevalence rates are a reflection of low dietary quality. Typical diets in the study region are sufficient in terms of calories but insufficient in terms of micronutrients [45]. Unlike urban Kenya, where changing retail environments contribute to a higher consumption of processed foods [46], rural households still derive most of their calories from unprocessed foods, partly from subsistence production. However, starchy staple foods often dominate the diets with too little quantities of fruits, vegetables, and animal products to guarantee sufficient micronutrient intakes. Individual-level DB was observed in male and female adults. Interestingly, prevalence rates were somewhat higher in male adults than in female adults. This is noteworthy, as male adults are rarely covered in dietary and nutrition surveys, nor in most nutrition interventions.

In children, individual-level DB problems were only observed in 1% of the cases. These low child DB rates are due to the fact that child overweight is not yet very widespread in the study region. Small children in rural Kenya suffer from various forms of undernutrition and micronutrient deficiencies, but their anthropometric measures rarely exceed the cut-off points for overweight or obesity.

Beyond individual-level DB, household-level DB problems have been analysed. Previous research on household-level DB had used different indicators of child undernutrition, which makes comparison and implications for policy response difficult. The most commonly used indicator of child undernutrition is stunting, which is a chronic condition. Household-level DB was found in 13% of the households when using child stunting. The prevalence of DB households was even higher (17%) when using micronutrient deficiency as the indicator of child undernutrition. When using child underweight and wasting, both more transient conditions, the prevalence of DB households is lower (< 3%), which is due to the fact that relatively few children in the study area suffer from underweight and wasting. Households with better educational levels are somewhat less affected by DB problems than households with less education, as one would expect.

Up till now, DB has been primarily described as an urban problem that is associated with more sedentary lifestyles, westernization of diets, and food environments that are increasingly dominated by modern retailers and fast food restaurants [3, 5, 14, 18, 22, 47]. The results reported here suggest that DB is also a problem in rural areas, where lifestyles and food environments are still more traditional. More research in other rural regions in Africa will be useful to test these results. Additionally, more evidence is needed to better understand the socioeconomic drivers of DB in different settings. This is particularly important in regard to the development of food and nutrition policies.

One question worth discussing is in how far the results from Western Kenya are representative for a larger region. Of course, the exact DB prevalence rates should not be extrapolated to other geographic regions, because every setting is different and because the sample size used here is relatively small. However, the sample households from Western Kenya were selected randomly. Most of them are involved in subsistence farming under rainfed conditions; small quantities of crop and livestock products are also sold in local markets. Farm sizes are very small, so agricultural income is often supplemented with off-farm economic activities. These are typical conditions for households in rural Africa. Hence, the general finding that various forms of DB problems exist can be cautiously generalized for rural Africa more broadly.

Another point worth discussing is the measurement of micronutrient malnutrition. In this article, micronutrient deficiency was calculated with individual-level food intake data for adults and children. This is different from earlier DB studies that had used biochemical data (blood samples) to detect specific micronutrient deficiencies [23–26]. The collection of biochemical data may lead to more precise estimates, but is also more costly and invasive for study participants [48]. The collection and use of food intake data may be a practicable alternative to include issues of micronutrient malnutrition more broadly into the DB literature.

## Conclusion

The main finding of this study is that double burden problems exist in rural Kenya. This is an important result also from a policy perspective, as it clearly underlines that an isolated focus on addressing calorie undernutrition is insufficient and may even be counterproductive for achieving broader nutritional improvements in rural households. Getting beyond the preoccupation with staple foods and instead promoting affordable access to dietary diversity will be important to address the different nutritional problems simultaneously.

Another key results of this study is that the prevalence rates of double burden problems vary and depend on how exactly double burden is defined and measured. Hence, comparisons across studies that build on different definitions have to be done with caution. There is need to harmonize definitions and compare different indicators in the same context to better understand the full picture of emerging double burden phenomena. In addition, further research on the socioeconomic drivers of DB problems in rural areas is needed; these drivers may not be the same as those in urban areas. Such research is an important precondition for developing suitable nutrition policies and interventions.

## Supplementary information

**Additional file 1:** Questionnaire of Household Survey 2016

**Additional file 2: Table S2.** Estimated average requirements (EAR) for micronutrients

**Additional file 3: Table S3.** Nutritional status of adults and children in the sample

**Additional file 4: Table S4.** Prevalence of micronutrient deficiencies in adults and children

**Additional file 5: Table S5.** Characteristics of adults, female and male, with and without individual-level DB

**Additional file 6: Table S6.** Characteristics of children with and without individual-level DB

**Additional file 7: Table S7.** Prevalence of household-level DB within different subsamples

**Additional file 8: Table S8.** Characteristics of households with and without household-level DB

## Data Availability

The datasets used and/or analysed during the current study are available from the corresponding author on reasonable request.

## Abbreviations

BAZ: BMI-for-age Z-score
BMEL: German Federal Ministry of Food and Agriculture
BMI: Body mass index
DB: Double burden of malnutrition
EAR: Estimated average requirement
HAZ: Height-for-age Z-score
n: number of observations
SD: Standard deviation
WAZ: Weight-for-age Z-score
WHO: World Health Organization
WHZ: Weight-for-height Z-score
