# Supplementary material for: Various forms of double burden of malnutrition problems exist in rural Kenya
Source: BMC Public Health. 2019 Nov 21;19:1543. doi: 10.1186/s12889-019-7882-y (PMC6873738; doi:10.1186/s12889-019-7882-y)
Supplement: Supplementary file 1 — Additional file 1: Questionnaire of Household Survey 2016 [file 12889_2019_7882_MOESM1_ESM.pdf]

Questionnaire number (adda_hhid) ____________________

Georg-August-Universität Göttingen

Africa Harvest BIOTECH FOUNDATION INTERNATIONAL

UNIVERSITY OF NAIROBI

HOUSEHOLD SURVEY 2016

AGRICULTURE AND DIETARY DIVERSITY IN AFRICA: AN APPLICATION OF RANDOMISED CONTROLLED TRIALS IN KISII AND NYAMIRA, KENYA.

Goettingen University-Germany, University of Nairobi-Kenya and Africa Harvest Biotech Foundation International (Africa Harvest) are carrying out a research on different aspects of agricultural development. We are currently doing a survey which aims to provide more understanding about farmers' production and marketing decisions, and nutrition and health status. Your participation in answering these questions is very much appreciated. Your responses will be **COMPLETELY CONFIDENTIAL** and will only be used for research purpose. If you indicate your voluntary consent by participating in this interview, may we begin?

MODULE 0 – HOUSEHOLD ID 3

TARGET PERSON: GROUP MEMBER OR HOUSEHOLD HEAD 4

MODULE 1: HOUSEHOLD DEMOGRAPHIC INFORMATION 4

MODULE 2: LAND HOLDING IN ACRES 5

MODULE 3: NON-LABOUR PURCHASED INPUT USE 7

MODULE 4: CROP UTILIZATION 9

MODULE 5: LABOUR INPUTS 10

MODULE 6: VARIETY/BREED AWARENESS AND UP-TAKE 11

MODULE 7: VARIETY/BREED ATTRIBUTES, KNOWLEDGE & PERCEPTION 12

MODULE 8: LIVESTOCK PRODUCTION AND MARKETING 13

MODULE 9: HOUSEHOLD ASSETS 14

MODULE 11: OTHER SOURCES OF INCOME AND TRANSFER 15

MODULE 12: NON-FOOD EXPENDITURE 16

MODULE 13: INFORMATION ON CREDIT ACCESS 16

MODULE 15: ACCESS TO SOCIOECONOMIC INFRASTRUCTURE 16

MODULE 17: SHOCKS EXPERIENCENCED BY THE HOUSEHOLD 17

TARGET PERSON: GROUP MEMBER 18

MODULE 18: SOCIAL CAPITAL ENDOWMENT 18

MODULE 14: COMMUNITY OUTREACH METHODS 19

MODULE 19: SOCIAL NETWORKS 22

TARGET PERSON: PERSON RESPONSIBLE FOR FOOD PREPARATION 30

MODULE 20: HOUSEHOLD FOOD CONSUMPTION 30

TARGET PERSON: MOTHER OR CARETAKER OF CHILD BETWEEN THE AGE OF SIX TO 59 MONTHS 34

MODULE 21: CHILD QUESTIONNAIRE – ONLY ONE CHILD WILL BE CONSIDERED 34

1. TARGET PERSON: FIRT INDIVIUDAL 37

MODULE 22/1- FIRST INDIVIDUAL QUESTIONNAIRE (1) 37

MODULE 23/1 - DECISION MAKING 41

2. TARGET PERSON: SECOND INDIVUDAL 42

MODULE 22/1- SECOND INDIVIDUAL QUESTIONNAIRE (2) 42

MODULE 23/2 - DECISION MAKING 47

Questionnaire number (adda_hhid) ____________________

We are researchers from Göttingen University-Germany, University of Nairobi-Kenya and Africa Harvest Biotech Foundation International (Africa Harvest). We are conducting research that aims to improve the knowledge on agriculture-nutrition linkages in the African small farm sector. We are particularly interested in understanding the mechanisms through which farmers can effectively adopt agricultural technologies that may improve their nutrition and health. We are currently conducting the first round of the survey last year and now will do a follow-up round.

This informed consent is for smallholder farmers [like you] who belong to farmer groups and have engaged in farming activities during the last one year (October, 2015 to September, 2016). We are inviting you to participate in this research that mainly focuses on nutrition and health status of smallholder farmers in this area. We will ask you and some members of your household detailed questions on various topics related to agriculture, social networks, nutrition and health. We will also need to take measurements of the height and weight of selected adults and children below 5 years of age in your household. Your participation in this interview is entirely voluntary. Your responses will be treated with utmost confidentiality and the data will be used for research purposes only.

Do you have any questions that we need to clarify? [Make clarifications in case there are questions]If *No*, do you agree to take part in this survey, including the interviews and the measurements of adults and children?

If *Yes*let the potential respondent write name and sign below

Name____________________________________

Signature_________________________________

Questionnaire number (adda_hhid) ____________________

MODULE 0 – HOUSEHOLD ID

1	Household ID		8	County		12	**First visit date**	
2	Group ID		9	Sub-County		1=Interview completed 2= Interview partly completed 3= Specify		
3	Date of interview		18	Ward		14	**Enumerator Name**	
4	Start Time (24 Hr)		17	Division		13	**Second visit date**	
5	End time (24 Hr)		10	Village		1=Interview completed 2= Interview partly completed 3= Specify		
6	HH head Full Name		11	GPS Coordinates		**15**	**Enumerator Name 1**	
7	Cell phone number					**16**	**Enumerator Name 2**	

Questionnaire number (adda_hhid) ___________________

TARGET PERSON: GROUP MEMBER OR HOUSEHOLD HEAD

Respondent MEMID: ____

MODULE 1: HOUSEHOLD DEMOGRAPHIC INFORMATION *(reference period between 1st Oct 2015 and 30th Sep 2016)*

Household composition**:** *Please list all household members (All those who are under the care of household head in terms of food and shelter provision, and those who normally live and eat their meals together), starting with the household head.*

1	2	3	4	5	6	7	10	11	12	13	14
**MEMID**	**Name of the HH member**	**Gender** *M = 1* *F= 0*	**R/ship with HH head** (*Codes A*)	**Age in years**	**Years of formal education** (*Highest level attained*)	**Marital Status** *(Codes B)*	**# of months in the last 12 months [NAME] has been away from home**	**Main Occupation based on time spent** *(Codes D)*	**Household farm labour contribution** *(for those above 16 years of age in the upper category) (Codes E)*	**How many hours per day are dedicated to farm activities? (hr)**	**If you had a larger farm how many hours per day would be dedicated to farm acitivities?**
			**1**								

Code A
1= Head
2=Spouse
3=Son/daughter
4=Father/mother
5=Sister/brother
6=Grandchildren
7=Grandparents
8=Step children
9=Step parents
10 = Father/mother-in-law
11 =Sister/brother-in-law
12 = House girl
13 =Farm labourer
16=Nephew/Niece
14 = Other relative
15= Other Unrelated

Code B
1= Married-monogamous
2= Married polygamous
3= Single
4= Divorced/separated
5= Widow/widower

Code D
0= None
1= Farming (crop + livestock)
2= Casual labour on-other farm
3= Casual labour off-farm
4= Self-employed off-farm
5= Salaried employment (civil servant etc)
6=Student/school
77= Other (Specify)______

Code E
1= Part time
2= Fulltime
3=Does not work on farm

Questionnaire number (adda_hhid) ____________________

MODULE 2: LAND HOLDING IN ACRES *(period between 1st Oct 2015 and 30th Sep 2016)*

2.1. How much land do you own in acres? _________________

2.2. How much of your total land is under homestead? ________________

2.3. Do you have a title deed for your land? ____________Yes=1 (all land), No=0 (no land), Partly=3

**Land category**	**Short rain season (Oct-Nov 2015)**		**Long rain season (Mar-Apr 2016)**	
	Cultivated	Fallow	Cultivated	Fallow
1. Own land (A)				
2. Rented in (B)				
3. Rented out (C)				
4. Total irrigated land				

2.4. What is the average cost of renting land per acre (Ksh/per year)? __________

CODES FOR MODULE 3

Codes A

1 Maize
2 Rice
3 Sorghum
4 Millet
5 Cassava
6 KK 15 Beans
7 Other Field beans
8 Bananas
9 Cabbage
10 Cowpea
11 Groundnut
12 Soybean
13 Sweet potatoes
14 Orange Fleshed Sweet Potatoes (OFSP)
15 Black night shade
16 Sugarcane
17 Pineapple
18 Jute Mallow (Omutere)
19 Amaranthas leaves (Emboga)
20 Pumpkin leaves
21 Sukuma wiki (Kales)
22 Carrots
23 Passion Fruit
24 Irish potato
25 Bean leaves
26 Tea
27 Onion
29 Coffee
30 Napier grass
31 Avocado
32 Spider Plant
33 Vine Spinache
34 Pumpkin
35 Trees
36 Mangoes
37 Guava
38 Wheat
39 Paw Paw
40 Tomatoes
41 Loquat
42 Green grams
43 Tree Tomato
44 Strawberry
45 Spring Onion
46 Desmodium
47 Spinach
48 Arrow Roots
49 Green Peas
50 Physallis/Gooseberry
51 Corriander
52 Capsicum
53 Pepper
54 Grass
55 Butternut
56 Lemon
57 Beetroot
58 Cumcumber
59 Water melon
60 Tree Seedlings
61 Raspberry
63 Pyrethrum
64 Cowpea Leaves
77 Other__________________
78 Other__________________
79 Other___________________

Codes B

0. Local
1. Improved
2. Mixture

Codes C

1. Kilogram
2. Litre
3. 90 Kg bag (40 Gorogoro)
4. 50 Kg bag
5. 25 Kg bag
6. Gorogoro (2.25 kg)
7. Debe (18 kg/ 8 Gorogoro)
8. Wheelbarrow
9. Ox-cart
10. Bunch (bananas)
11. Piece/number
12. Not yet harvested (for perennials only)
13. Stools
14. Glass (250 gr)
15. Suckers
16. Bucket
17. Ml
18. Spoonful
19. 5 kg bag
20. 10 kg Bag
22. Yellow paper bag
23. Grams
24. Pick up
25. Trees
26. Green paper bag
27. Lines
28. Packet (250g)
29. Crates
30. Bundle
31. Handful
32. Cuttings
33. Vines
35. Lorry
36. Seeds
37. Bushes
38. 45kg bag
39. Bottle top
40. Seedlings
41. Tonne
42. 500 Ml glass
45. Cobs
46. Poles
47. Crop failure
48. Black paper bag
77 Other (specify)________

Questionnaire number (adda_hhid) ___________________

MODULE 3: NON-LABOUR PURCHASED INPUT USE (*1st Oct 2015 and 30th Sep 2016 planting seasons, record separately by plots*)

**1**	2	3	12	4	5	6			7			8	9			10			11	
**Plot Code (*Use alphabets in Cap*)**	Crop Grown ***A***	Land under crop (*acre*)	Intercrop (1=Yes; 0=No)	Number of trees	Crop variety **B**	Seed **C**			Fertiliser(planting) (Fill once for intercrops) **C**			Oxen/ tractor hire Cost	Farm manure (Fill once for intercrops) ***C***			Pesticides/herbicides ***C***			Crop output ***C***	
						Qty	units	Price /Unit	Qty	Units	Price /Unit	Ksh	Qty	unit	Price /unit	Qty	units	Price /unit	Qty	Unit
**Short Rains**																				
	KK 15 (6)																			
**Long Rains**																				
	KK 15 (6)																			
**Perennial Crops**																				

Questionnaire number (adda_hhid) ____________________

CODES FOR MODULE 4 (*period between **1st Oct 2015 and 30th Sep 2016***)

Codes A

1 Maize
2 Rice
3 Sorghum
4 Millet
5 Cassava
6 KK 15 Beans
7 Other Field beans
8 Bananas
9 Cabbage
10 Cowpea
11 Groundnut
12 Soybean
13 Sweet potatoes
14 Orange Fleshed Sweet Potatoes (OFSP)
15 Black night shade
16 Sugarcane
17 Pineapple
18 Jute Mallow (Omutere)
19 Amaranthas leaves (Emboga)
20 Pumpkin leaves
21 Sukuma wiki (Kales)
22 Carrots
23 Passion Fruit
24 Irish potato
25 Bean leaves
26 Tea
27 Onion
29 Coffee
30 Napier grass
31 Avocado
32 Spider Plant
33 Vine Spinache
34 Pumpkin
35 Trees
36 Mangoes
37 Guava
38 Wheat
39 Paw Paw
40 Tomatoes
41 Loquat
42 Green grams
43 Tree Tomato
44 Strawberry
45 Spring Onion
46 Desmodium
47 Spinach
48 Arrow Roots
49 Green Peas
50 Physallis/Gooseberry
51 Corriander
52 Capsicum
53 Pepper
54 Grass
55 Butternut
56 Lemon
57 Beetroot
58 Cumcumber
59 Water melon
60 Tree Seedlings
61 Raspberry
63 Pyrethrum
64 CowPea Leaves

77 Other__________________
78 Other__________________
79 Other___________________

Codes C

1. Kilogram
2. Litre
3. 90 Kg bag (40 Gorogoro)
4. 50 Kg bag
5. 25 Kg bag
6. Gorogoro (2.25 kg)
7. Debe (18 kg/ 8 Gorogoro)
8. Wheelbarrow
9. Ox-cart
10. Bunch (bananas)
11. Piece/number
12. Not yet harvested (for perennials only)
13. Stools
14. Glass
15. Suckers
16. Bucket
17. Ml
18. Spoonful
19. 5 kg bag
20. 10 kg Bag
22. Yellow paper bag
23. Grams
24. Pick up
25. Trees
26. Green paper bag
27. Lines
28. Packet (250g)
29. Crates
30. Bundle
31. Handful
32. Cuttings
33. Vines
35. Lorry
36. Seeds
37. Bushes
38. 45kg bag
39. Bottle top
40. Seedlings
41. Tonne
42. 500 Ml glass
45. Cobs
46. Poles
47. Crop failure
48. Black paper bag
77 Other (specify)________

Codes D

1. Farm gate
2. Village market
3. Main market
4. Collection center
77. Other (specify)_________

Codes E

1. Own bicycle
2. Bodaboda
3. Hired truck
4. PSV
5. Donkey/oxen
6. Walking
7. Own truck
8. Taxi
77 Other (sp.)
99. NA

Code F

1. Male household head
2. Female household head
3. Female spouse
4. Joint decision
5. Male spouse
77 Other (specify)___________

Questionnaire number (adda_hhid) ___________________

MODULE 4: CROP UTILIZATION *(in the period between 1st Oct 2015 and 30th Sep 2016)*

1	2		3		4		5		6		7		8		9	11	10	12	13	14
Crop **Code A** (Aggregated crop)	Total Crop Output (Enter the total crop output from the plots)		Consumption		Saved as seed		Gift, tithe, donations, paid as wages		Stored		Quantity sold		Price		Point of most sales ***D***	Main Mode of transport **E**	Travel time to the point of sale (minutes)	Who mostly decides revenue use? **F**	Who mostly decides technology use e.g. variety **F**	Who mostly decides how much of the total output is consumed by the household? **F**
	Qty	*Unit* ***C***	*Qty*	*Unit* ***C***	*Qty*	*Unit* ***C***	*Qty*	*Unit* ***C***	*Qty*	*Unit* ***C***	*Qty*	*Unit* ***C***	*Ksh*	*Unit* ***C***						
**Short rain**																				
KK 15 (6)																				
**Long rain**																				
KK 15 (6)																				
**Perennial crop**																				

Questionnaire number (adda_hhid) ____________________

4.1 How easily can you access the market for sale of your produce (crop and or livestock)? *(Circle the applicable)*
1. Very easy 2. Easy 3.Difficult 4. Very difficult

4.2 Rank three most important market access constraints, if there exists any (***Prompt Codes G below***) 1.________________2.________________3.______________
Codes G:1. Poor infrastructure 2. Distant markets 3. Poor market prices 4. Cheating on quality standards/weighing scales 5.Lack of contracts or reliable buyers 6.Exploitative middlemen
77. Other (specify): ________________

4.7 In the last one year did you order for: 1 KK15 ____ (1.Yes; 0. No); 2. Kuroiler chicken ____ (1.Yes; 0. No);

If the respondent is not growing KK 15 beans, skip to module 5

4.3 How easily can you market your KK15/beans? *(Circle the applicable)*
1. Very easy 2. Easy 3. Difficult 4. Very difficult

4.4 What is the **MAIN REASON** for your answer in 4.3 above *(Circle the applicable)*
1. Distance to market 2. Colour of beans 3. Prices 4. Yield 5. Taste 6. Pest and disease resistance 7. Cooking quality 8. Nutritional value
77. Others (specify)___________ -99 N/A

4.5 When did you first order the KK15 bean seed? Date _________________ Month_____________

4.6 When did you receive KK 15 seeds for the first order? Date_________________ Month_________________

MODULE 5: LABOUR INPUTS (*01.Oct 2015 to 30. Sept 2016 planting seasons, record* ***total man hours*** *worked by plot)*

1	2	3	4	5
**Plot code**	Plot size in acres	Plot manager *(F=0, M=1; Joint=3)*	Ploughing & harrowing Planting & thinning Applying fertiliser, Pesticide application (1st and 2nd) Weeding (1st and 2nd) Harvesting /Threshing/shelling/bagging	
			Family	Hired
**Short Rains**				
A				
B				
C				
D				
E				
F				
G				
H				
**Long Rains**				
A				
B				
**C**				
D				
E				
F				
G				
H				

5.6 What is the average daily wage rate for men and women in this village? Men________ Ksh/per day   Women__________Ksh/per day

5.7 Given all the family labour (manual) available in your household, what is the **maximum land size** in acres that you could potetntially cultivate and keep under livestock? ________________________________

MODULE 6: VARIETY/BREED AWARENESS AND UP-TAKE

	1	2	3	14	4	15	5	6	7	8	9	10	11	12	13
	New breed/variety/technologies	Have you ever heard of this variety/breed? *(1=Yes; 0=No) If No skip to the next technology*	**Main** source of information on the new variety/breed? **Codes A**	How easily can you obtain information from **main source**? **Code D**	Have you ever planted /kept this variety/breed? *(1=Yes; 0=No) If NO, skip to 5*	If yes, name the most important reason for adopting **Code E**	If No to Q4, what was the main reason? **Codes C** *Then Skip to Q10*	What was the **main** source of breed kept/variety planted that year? **Codes B**	Number of seasons the variety has been planted, **since first** planting?	Number of years /variety/breed has been planted/kept	If you did not plant this variety/keep breed in 2016 what was the main reason? **Codes C**	Will you plant the variety/ keep the breed in future? *(1=Yes; 0=No, 88=don't know) if Yes skip to Q12*	What is the main reason? **Codes C**	Are you aware of the nutritional value of this variety or breed? (yes = 1, No = 0)	If yes to Q12 what was the source of information? **Code A**
3	*Kuroiler chicken*														
4	*Beans(KK15)*														

Code A	Code B	Code C	Code D	Code E
1= Farmer Coop/Union<br>2= Farmer group<br>3= Extension staff/office<br>4= Other farmers (neighbours/relative)<br>5= Market (e.g. Agro vet/stockist)<br>6= Radio programs<br>7= Research centre (trials/demos) (*name* ___<br>8= NGO/CBO (*name* ______<br>*9= Health centre/Practitioner*<br>77= Other*(specify ______)*	1= NGO free *(name _______)*<br>2= NGO subsidy *(specify______)*<br>3= Extension staff demo plots<br>4= Other farmers<br>5= Market (Agrovet/local trader/stockist)<br>6= Farmer group/coop<br>7=Agricultural association/training centre<br>77= Other*(specify _________)*	1= Seed not available<br>2=Day old chicks not available<br>3=Lacked cash to buy seed/DOCs<br>4= Lacked credit to buy seed/DOCs<br>5= Prefer other varieties/breeds<br>6=Susceptible to diseases/pests<br>7=Poor taste<br>8=Low yielding/lays fewer eggs<br>9=Late maturing /longer maturity period<br>10=Low market prices/demand<br>11=High input requirements<br>12=Limited land to experiment/plant<br>13= Limited information<br>77= Other*(specify ______)*	1=Very easy<br>2= Easy<br>3=Difficult<br>4= Very difficult	1= Seed easily available<br>2= Day old chicks easily available<br>3= Availabiliy of cash to buy seed/DOCs<br>4= Availability of credit to buy seed/DOCs<br>5= Preference KK 15/Kuroiler<br>6= Resistance to diseases/pests<br>7= Good taste<br>8= High yielding/lays many eggs<br>9= Early maturing /shorter maturity period<br>10= High market prices/demand<br>11= Lower input requirements<br>12= Adequate land to experiment/plant<br>13= Sufficient information<br>14= Seed/DOC Subsidy<br>77= Other*(specify ______)*

MODULE 7: VARIETY/BREED ATTRIBUTES, KNOWLEDGE & PERCEPTION

Instructions: Only ask the following questions to farmers who have ever heard or grown or kept the new technologies (listed below).

If *Yes*, ask for his/her perception of the performance of the technology (ies) against the listed attributes compared to his/her preferred local variety /breed. *Please mark the respondent's response with a* ***tick*** *in the appropriate cells below. If No, skip to the next module.*

	1	2				3			
		Kuroiler chicken				*Beans (KK15)*			
	Do you know the attributes of the following technologies? *Yes=1 No=0*	_________ *If No Skip to the next technology, IF Yes ask for the attributes*				_________ *If No Skip to the next technology, IF Yes ask for the attributes*			
	Technology attributes	*Better*	*Worse*	*No difference*	*Don't know*	*Better*	*Worse*	*No difference*	*Don't know*
1	Early maturity								
2	Yield								
3	Pest and disease resistance								
4	Marketability (demand)								
5	Cost of planting materials								
6	Market price received								
7	Cost of day old chicks								
8	Taste								
9	Lays more eggs								

7.8 How easily can you market your Kuroiler chicken*? (Circle the applicable)*
1. Very easy   2. Easy   3.Difficult   4. Very difficult   88. DNK

7.9 How easily can you market your Kuroiler eggs*? (Circle the applicable)*
1. Very easy   2. Easy   3.Difficult   4. Very difficult   88. DNK

7.10 What is the **MAIN REASON** for your answer in 7.8 above *(Circle the applicable)*
1.Early maturity   2.Pest and disease resistance   3.Maketability   4.Market price received   5. Cost of day old chicks   6. Taste   7. Lay more eggs   77.Others (specify)   -99 N/A

7.11 What is the **MAIN REASON** for your answer in 7.9 above *(Circle the applicable)*
1. Taste   2 Price   3. Size   4. Colour of the yolk   -99 N/A

MODULE8: LIVESTOCK PRODUCTION AND MARKETING

8.1 For the last **12 months *(1st Oct 2015 and 30th Sep 2016)***, please give details of revenue and cost of livestock production?

(Please include all animals on the farm last year also those that were later sold or died) If no livestock is owned skip to next module)

	1	2a	2b	3a	3b	4a	4b	5a	5b	6	7	8	9	10	11	12	13
	Animal species	Stock at the beginning of the period (01.Oct.2015) (*If 0, skip to the next*)		Changes over the years: Home consumption		Changes over the years: Sales		Stock at the end of 30.Sep.2016		Cash expenditures between 10/15 and 9/16 Value in Ksh				Who decides sale?	Who decides revenue use?	Who decides technology use e.g. breed	Who mostly decides how much of the total output is consumed by the household?
		**Unit**	**Ksh**	**Units**	**Ksh**	**Units**	**Ksh**	**Units**	**Ksh**	**Veterinary treatment**	**Feed**	**Hired labor**	**Others, specify:**	**B**	**B**	**B**	**B**
1	Dairy cows/calves																
2	Cow/calves																
3	Goat																
4	Sheep																
5	Kuroiler/chicks																
6	Other chicken/chicks																
7	Donkeys																
8	Pigs																
9	Rabbits																
10	Ducks																
77																	
78																	

8.2 For the last **12 months *(01. Oct 2015 to 30. Sep 2016)***, please give details of production and revenue of the following livestock products?

	**1**	2		3		4		8		5	6	7
	**Animal product/services**	Quantity produced: Qty	Unit **A**	Quantity sold: Qty	Unit **A**	Quantity Consumed: Qty	Unit **A**	Other, specify: Qty	Unit **A**	Price per unit	Who decides sale? **B**	Who decides revenue use? **B**
1	Milk											
6	Kuroiler Eggs											
2	Other Eggs											
7	Kuroiler Manure											
3	Manure											
4	Honey											
5	Hide											
77	Others specify_________											

Code A: *1=Kilogram, 2=Litre, 3=90 Kg bag, 4=50 Kg bag, 5=25 Kg bag, 6=Gorogoro, 7=Debe, 8=Wheelbarrow, 9=Ox-cart, 10=Bunch (bananas), 11=Piece/number, 50=Tray, 77=Other (specify)* ________

Code B: *1=Male household head, 2= Female household head, 3=Female spouse, 4=Joint decision, 77= Others (specify)*___________

MODULE 9: HOUSEHOLD ASSETS *(Prompt for each item as listed below)*

9.1 As at September 2016, how many of the following items did the household own that are in **usable/repairable** condition?

To estimate the value ask the respondent how much they would be willing to buy the item in its current state if it were being sold to them

	ASSET	Total Quantity	Estimate total current value of the asset(s) if you were to buy it in its current state		ASSET	Total Quantity	Estimate total current value of the asset(s) if you were to buy it in its current state
**1**	Tractor			**2**	Slasher		
**3**	Car/Van			**4**	Axe		
**5**	Pickup			**6**	Panga		
**7**	Motorcycle			**8**	Hoes/Jembes		
**9**	Bicycle			**10**	Spades/shovel		
**11**	Television			**12**	Chemical spray pump		
**13**	Radio			**14**	Treadle pump		
**15**	Mobile Phone			**16**	Powered water pump		
**17**	Refrigerator			**18**	Mosquito net		
**19**	Solar panels			**20**	Greenhouse		
**21**	Generator			**22**	Water tank		
**23**	Chaff cutter			**24**	Store for farm produce		
**25**	Ploughs for tractor			**26**	Lanterns		
**27**	Reaper			**28**	Main house		
**29**	Ox-plough			**30**	Wheelbarrow		
**31**	Cart			**32**	Computer/laptop		
**33**	Livestock Kraal			**34**	Biogas digesters		
**35**	Other(specify_______			**36**	Other(specify_______		
**37**				**38**			

MODULE 11: OTHER SOURCES OF INCOME AND TRANSFER

11.1 Do you have off farm employment? ______________ (*1=Yes; 0=No*) *If NO, skip to 11.2.*

Please prompt the codes to make sure nothing is forgotten					
1	2	3	4	5a	5b
MEMID	Type of Occupation<br>**A**	Average Number of days worked per month 10/15 – 9/16	Average Number of months worked per year 10/15 – 9/16	Earning per unit	
				Ksh	**B**

***Code A**: 1= Agricultural labour (casual+permanent), 2= Casual labour (non-agricultural), 3= Salary (Permanent non-agricultural employment)*
***Code B**: 1= Day, 2= Month, 3= Year, 4= Lump sum, payment, 77= Other, specify: __________________________________*

11.2 Do you have any other sources of income? __________ (*1=Yes; 0=No*) *If NO, please probe and skip to 12.*

	Please prompt the codes to make sure nothing is forgotten			
	1	2	3	4
	Categories	Code	Type of occupation	Amount /value received between Oct15/ Sept 16/ *for small businesses ask for profit (+) losses (-)*
1	Remittances/gifts/transfers/food aid	1		
2	Pension	2		
3	Small business	1	Brick making	
		2	Carpentry	
		3	Construction	
		4	Grain mill	
		5	Handicrafts	
		6	Beverage, local brew	
		7	Sales in shop, petty trade	
		8	Transport	
		77	Other, specify__________________________	
4	Sales of forest products	9	Sale of wood and charcoal	
		10	Sale of wild nuts/fruits	
5	Other agric. Income	11	Sale of crop residues	
		12	Leasing out land	
		13	Renting out oxen for ploughing	
		14	Hiring out machinery services to other farmers	
		15	Dividends (T-bills, bonds, shares)	
		16	Tea bonus	
6	Other	35	Betting	

MODULE 12: NON-FOOD EXPENDITURE

Consider the **last year (Oct 15 - Sept 16)** generally how much has your HH spent on the items listed in a typical year (see specification indicated for each item)?

		1	2
		Read out: Please exclude ***Business Expenditures***	How much did your household spend on [ITEM/SERVICE] during the last year (Oct. 15 – Sept 16)?
		Enter 88, if respondent does not know.	Value in Khs
**Non-food**	1	Rent (housing)	
	2	Personal care supplies	
	3	Clothes, shoes and bags, accessories	
	4	Detergent/washing powder	
	5	Electricity	
	6	Other non-food	
**Transportation + communication**	7	Fuel, maintenance, insurance, and tax for motorbike/car	
	8	Public transport	
	9	Airtime (incl. MPESA)	
	10	Other transportation, communication	
	11		
	12		
**Education**	13	School fees, books, Student's dress/uniform, Tuition and rental fee	
	14	Other cost of schooling	
	15		
	16		
**Health**	17	Medicine, doctor fees	
	18	Other health cost	
	19		
	20		
**Social**	21	Celebration and funeral cost	
	22	Recreation and entertainment	
	23	Contributions (eg. Church, groups)	
	24	Tobacco (incl. snuff and miraa)	
	25	Insurance (eg. Car, life, health)	
	26	Remittances transferred to other HH	
	27	Other social cost	
	28		
	29		

MODULE 13: INFORMATION ON CREDIT ACCESS

13.1 Could you obtain credit if you needed it for the purpose of operational agricultural expenses (e.g. buying fertilizer paying for labour etc.)? ____________________*1=Yes, 0=No*

13.2 During the last ***12 months (Oct15 to Sep16)***, have you or any other household member received any credit to buy inputs, or received inputs on credit?_________________*1=Yes, 0=No*

13.3 If yes to 13.2, how much did you receive in Ksh? (___________________) (*Include the value of inputs if inputs are provided on credit*)

13.4 How much went into purchasing inputs? (_______________) (*Include the value of inputs if inputs are provided on credit*)

MODULE 15: ACCESS TO SOCIOECONOMIC INFRASTRUCTURE

1	2	3
**Social facilities**	**Distance to the nearest (km)**	**Most frequently used means of transportation to the facility** *(Use codes A below)*
1. Murram road		
2. Tarmac road		
3. Village market		
4. Main Agricultural input market		
5. Main agricultural product market		
6. Health centre		
7. Agric. Extension agent		

Code A: Means of transport Codes
1= Bicycle; 2= Motorbike; 3= Car; 4= Walk;
77= Others, (specify) ________

Questionnaire number (adda_hhid) ____________________

MODULE 17: SHOCKS EXPERIENCENCED BY THE HOUSEHOLD

	1	2	3	4
	**Please answer the following questions accordingly**	Did you experience [NAME OF SHOCK] in the last 12 month?<br>*1=Yes, 0=No*<br>*If No Skip to the next shock*	If yes, how many times has it occurred?	What was the intensity of the last shock to this household?<br>1= Severe<br>2= Moderate<br>3= Mild
	**Climatic shocks**			
1	Drought			
2	Floods			
3	Frosts			
4	Hailstorm			
	**Biological shocks**			
5	Pests or diseases that affected crops before harvest			
6	Pests or diseases that led to storage losses			
14	Loss of livestock			
	**Economic shocks**			
7	Large increase in agricultural input prices			
8	Large decrease in agricultural output prices			
9	Large increase in food prices			
	**Other shocks**			
10	Loss of family member			
11	Job loss			
12	Acute illness			
77	Other, specify____________________________			

Questionnaire number (adda_hhid) ___________________

TARGET PERSON: GROUP MEMBER

Respondent MEMID: _________________________

MODULE 18: SOCIAL CAPITAL ENDOWMENT

18.1 List all the groups you belong to (*Start with the sampled group)*

1	2	3	4	8	9	10	11
Group Name	Group Type<br><br>**A**	Please name the most important group function<br>**B**	Year joined	Participation in meetings in the reference period (Oct 15/Sep16)<br>**D**	Your own role in the group<br><br>**E**	Did the group receive any agricultural training during the reference period (Oct15/Sept16)<br>Yes= 1; N0= 0	Who offered the training?<br><br>AH= 1; Other= 0, AH+other= 2, DNK= 88

In case sampled group was not named in table above, answer 18.9, 18.10 and 18.11, otherwise skip to 18.2.

18.9 Are you still a member of the sampled group (NAME)? _______________ (Yes= 1; No= 0)

18.10 If no: Please shortly explain why you left the group: ____

18.11 In case, you received agricultural training from Africa Harvest in the sampled group: Who mostly informed you about the single training session (time and place)? _____________________________________ *(1= Group leader, 2= Other members, 3= Extension officer, 4= He was not informed, 77= Other, specify: _______________, -99= N/A)*

18.2. Do you personally exchange information with the local authorities/gov't agencies? ______ (1= Yes; 0= No)

18.6 Do you hold any of the other following positions*:* ____ *(Multiple answers possible)*

(0=No, 1= Village chief, 2= Village elder, 3= Nyumbakumi, 4= Religious leader, 77= Other_______________)

18.7 Are you a close relative to one of the mentioned positions *(1=Yes; 0=No)* ___________

18.8 If yes: Name position and relative: a. Position: _________ (Code 18.6) Relative _______ F

b. Position: _________ (Code 18.6) Relative _______ F

Codes A
1. Farmer cooperative
2. Farmers group
3. Women`s association
4. Youth association
5. Faith-based association/group
6. Funeral association/insurance group
7. Savings and credit group
8. Community based organization
9. Water users association
10. Informal labour sharing group
11. Widow/ widower
12. Family group
77. Other (______)

Codes B
1. Produce marketing
2. Input access or marketing
3. Seed production
4. Farmer research
5. Savings and credit
6. Welfare/funeral activities
7. Tree planting/Nursery
8. Soil & Water conservation
9. Faith-based organization
10. Input credit
77. Other (______)

Codes D
1. Always
2. Sometimes
3. Rarely
4. Never

Codes E
1. Official
2. Ex-official
3. Ordinary member

Code F
1. Parent
2. Spouse
3. Child
4. Brother/sister
5. Grandparent
6. Grandchild
7. Nephew/Nice
8. Uncle/Aunt
9. Cousin
10. Mother/father in low
11. Brother/Sister-in law
12. Other relative
13. Neighbour
14. Friend
15. Fellow villager
16. Attend same church/mosque
17. Business colleague
77. Other, specify _________

18.12 Please cross the correct answer:

1= Disagree strongly, 2= Disagree a little, 3= Neither agree nor disagree, 4= Agree a little, 5= Agree strongly, 6= Don't know

I see myself as someone who …		1	2	3	4	5	6
1	…finds it important to help the people nearby, to care for their wellbeing						
2	…Is reserved						
3	…is generally trusting						
4	…finds it important to think up new ideas and be creative, to do things on my own way						
5	…who's happiness depends on people around myself						
6	…is outgoing/social						
7	…tends to find fault with others						
8	…finds it important to maintain harmony within ones group						
9	…would always sacrifices one's self interest for the group						
10	…would stay in a group if they needed me, even if I were not happy with the group						

MODULE 14: COMMUNITY OUTREACH METHODS

14.1 How many visits or contacts with extension agents did you have during the last one year (Oct/2015 to Sep/2016)

1= Government extension___________ 2=NGO __________ 3=Private__________

If NO/ZERO to all in 14.1, skip to 14.7

14.2 State the **most important** type of agricultural information that you sought/received from the extension agent in the period between Oct 2015 and Sep 2016 ***(use codes B below)***

1= Government extension_________ 2=NGO __________ 3=Private__________

Code B

1. New crop varieties/breeds
2. Agric. credit/insurance
3. Field pest and disease control
4. Input quality
5. Input availability
6. Input markets and prices
7. Output markets and prices
8. Soil and water management
9. Crop rotation/ Minimum tillage
10. Safe use of pesticides
11. Adaptation to climate change
12. Postharvest handling
13. Collective action/farmer organization
14. Livestock production
15. Tree planting
77. Other…………
78. Other…………

14.3 In general, how would you rate your satisfaction with the information that you received? (***Use codes C below***)
1= Government extension_________ 2=NGO __________ 3=Private__________

Code C
1=Very unsatisfied 2 =Unsatisfied 3=Satisfied 4=Very satisfied

Questionnaire number (adda_hhid) ____________________

14.7 Characteristics of the extension officers

Section 14.7 is <u>applicalble only</u> if the respondent received training from Africa Harvest (see section 18.1 question 11 above)

14.8 I would like to know the gender of the officer who conducted trainings in your group _________ *1=Male, 0=Female*

14.9 What training did you attend? *(Please circle all applicable)* [1] Agriculture [2] Nutrition [3] Marketing [99] None

If NONE, please skip to 19.

14.10 What training session did you find **most important**? *(Please circle one)*

[1] KK 15 husbandry [2] Kuroiler husbandry [3] KK 15 post harvest management [4] Kuroiler health management [5] KK 15 marketing

[6] Kuroiler marketing [7] Malnutrition [8] Balanced diet [9] Food Safety

14.11 Could you please rate the officer with regard to:

		1	2
		Aspect	Could you please rate the officer with regard to: (1= Poor, 2=Fair, 3=Good, 4=Excellent)
**Human Relations**	1	Integration with farmers	
	2	Influence in adopting the technologies	
	3	Display of understanding of farmers situations	
	4	Keeping promises	
**Comunication skills**	5	Clarity of concepts	
	6	Detailed in delivering their message	
	7	Encouragement of exchange of experiences during training	
	8	Use of feedback systems	
	9	Provision of follow-up sessions	
	10	Encouragement of questions and enquires	
	11	Good listening skills	
	12	Provision of relevant response to questions	
	13	Use of communication aids e.g. flip charts	
	14	Use of appropriate languages to enhance understanding	
**Role performance**	15	Frequency of farm visit	
	16	Availability to farmers	
	17	Knowledge of nutrition enhancing technologies	
	18	Knowledge of good farming practises	
	19	Encouragement of farmers to share information with others	
	20	Time management	

Questionnaire number (adda_hhid) ____________________

MODULE 19: SOCIAL NETWORKS

Code A

**1**	Parent	**11**	Brother/Sister-in law
**2**	Spouse	**12**	Other relative
**3**	Child	**13**	Neighbour
**4**	Brother/sister	**14**	Friend
**5**	Grandparent	**15**	Fellow villager
**6**	Grandchild	**16**	Attend same church/mosque
**7**	Nephew/Nice	**17**	Business colleague
**8**	Uncle/Aunt	**77**	Other, specify___
**9**	Cousin		
**10**	Mother/father in low		

**19.1. General information about each group member**									
1	2	3	4	5	6	7	8	9	32
MEM ID	Name of the group member	Do you know NAME? *(1=Yes; 0=No), (-99=N/A)*	Please specify your relationship to NAME<br>**A**	Is NAME's plot bordering yours? *(1=Yes; 0=No)*	Do you know the kind of crops NAME grows?<br>*(1=Yes; 0=No)*	Do you know the kind of livestock NAME keeps? *(1=Yes; 0=No)*	Did you lend or borrow any of the following production means from NAME between Oct15 and Sept16?<br>0=no<br>1=lend<br>2=borrow<br>3=lend &borrow		Do you exchange / share food items?<br><br>*(1=Yes; 0=No)*
							Seeds	Agric. Produce	
		1							
		2							
		3							
		4							
		5							
		6							
		7							
		8							
		9							
		10							
		11							
		12							
		13							
		14							
		15							
		16							
		17							
		18							
		19							
		20							
		21							
		22							
		23							
		24							
		25							
		26							
		27							
		28							
		29							
		30							
		31							
		32							
		33							
		34							
		35							
		36							
		37							
		38							
		39							
		40							

Questionnaire number (adda_hhid) ____________________

**19.1. General Information about each group member**						
**1**	**2**	**10**	**11**	**12**	**16**	**13**
MEM ID	Name of Group Member	If you suddenly needed money, would you ask NAME to lend it to you? (*1=Yes; 0=No),*	Inside of this group: who are the farmers who would adopt new cropping technologies **first**? *Please mark with X*	Inside of this group: who are the farmers who would adopt new livestock technologies **first**? *Please mark with X*	Have you visited NAME between Oct15/Sep16 ? (*1=Yes;0=No)*	Have you talked to NAME between Oct15/Sep16? *(1=Yes; 0=No), if no cross name out and skip to next person*
		**1**				
		2				
		3				
		4				
		5				
		6				
		7				
		8				
		9				
		10				
		11				
		12				
		13				
		14				
		15				
		16				
		17				
		18				
		19				
		20				
		21				
		22				
		23				
		24				
		25				
		26				
		27				
		28				
		29				
		30				
		31				
		32				
		33				
		34				
		35				
		36				
		37				
		38				
		39				
		40				

Code A

1	Very often
2	Often
3	Sometimes
4	Rarely

Code C

1	Preparation of meals
2	Choice of products
3	Nutritional state of children
4	Quantity of food
5	Composition of meals
6	Content of nutrition training
7	Balanced diet
77	Other, specify_______

**19.1. Specific interaction within the farmer group** ***(remind respondent of nutrition definition)***							
**1**	**2**	**14**	**17**	**18**	**19**	**23**	**24**
MEM ID	Name of the Group Member	How often did you talk with NAME between Oct15/Sep16? **A**	Did you share information on nutrition with NAME? *(1=Yes;0=No) If no skip to 19*	Name the specific nutrition topic you mostly talked about with NAME **C**	Did you share information on agriculture with NAME between Oct15/Sept16? *(1=Yes; 0=No), if no, skip to next person*	Did you share information on Kuroiler chicken with NAME? *(1=Yes; 0=No)*	Did you share information on beans (KK 15) with NAME? *(1=Yes; 0=No)*
1							
2							
3							
4							
5							
6							
7							
8							
9							
10							
11							
12							
13							
14							
15							
16							
17							
18							
19							
20							
21							
22							
23							
24							
25							
26							
27							
28							
29							
30							
31							
32							
33							
34							
35							
36							
37							
38							
39							
40							

PLEASE USE THE 8 DIGIT MEMID (SEE GROUPLIST)

25. Who do you think is the **most informed person** among the group members concerning **nutrition** information?

_____________________ MEMID

26. Who do you think is the **most informed person** among the group members concerning **agricultural** information?

_____________________ MEMID

27. Why did you decide to become a group member? Give reason ____

28. Were you asked by another member to join the group ________ *(Yes=1, No=0)*

29. If yes: By whom? _________ ? MEMID

30. Have you introduced new people to this group? _____ *(Yes=1, No=0)*

31. If yes: Whom? MEMID__________ MEMID__________ MEMID__________

33. Please consider a situation where an organization offers agricultural training to your group.

However, the agricultural extension officers will only train 3 persons of your group. These persons are supposed to forward the information to the group.
Who do you think are the 3 most suitable persons of your group for this purpose?

MEMID__________ MEMID__________ MEMID__________

34. Do you like this approach or would you prefer that all group members should be able to participate in the training?
1=only 3 persons, 2= all group members

35. Now imageine the same situation, but the organization offers nutrition training to your group.

However, the NGO will only train 3 persons of your group,
Who do you think are the 3 most suitable persons of your group for this purpose?

MEMID__________ MEMID__________ MEMID__________

36. Do you like this approach or would you prefer that all group members should be able to participate in the training?
___ 1=only 3 persons, 2= all group member

19.2. SPECIFIC INTERACTIONS OUTSIDE THIS COMMON INTEREST GROUP

19.2.1 Please name the persons outside of your common interest group you most frequently exchanged information about nutrition between Oct15/Sept16. Please name a maximum of 5 persons: OUT ID 40_____________ OUT ID 40_____________ OUT ID 40_____________ OUT ID 40_____________ OUT ID 40_________________

19.2.2 Please name the persons outside of your common interest group you most frequently exchanged information about agriculture between Oct15/Sept16. Please name a maximum of 5 persons: OUT ID 40_____________ OUT ID 40_____________ OUT ID 40_____________ OUT ID 40_____________ OUT ID 40_________________

ID Section								
1	2	3	4	6	9	10	12	**11**
OUT ID	Name	NAME's gender *Male=1, female=0*	Please specify your relationship to NAME **A**	How often did you talk with NAME between Oct15/Sep16? **B**	Did you lend or borrow any of the following production means from NAME between Oct15 and Sept16? 0=no 1=lend 2=borrow 3=lend &borrow		Do you exchange/ share food items? *(1=Yes; 0=No)*	If you suddenly needed money, would you ask NAME to lend it to you? *(1=Yes; 0=No)*
					Seeds	Agric. Produce		
40								
40								
40								
40								
40								
40								
40								
40								
40								
40								

12. Who do you think is the **most informed person** among the ones named concerning **nutrition** information? 40______________________OUT ID

13. Who do you think is the **most informed person** among the ones named concerning **agriculture** information? 40______________________OUT ID

Code A

**1**	Parent	**6**	Grandchild	**11**	Brother/Sister-in law	**16**	Attend same church/mosque
**2**	Spouse	**7**	Nephew/Nice	**12**	Other relative	**17**	Business colleague
**3**	Child	**8**	Uncle/Aunt	**13**	Neighbour	**77**	Other, specify___
**4**	Brother/sister	**9**	Cousin	**14**	Friend		
**5**	Grandparent	**10**	Mother/father in low	**15**	Fellow villager		

Code B

**1**	Very often
**2**	Often
**3**	Sometimes
**4**	Rarely

Questionnaire number (adda_hhid) ___________________

TARGET PERSON: PERSON RESPONSIBLE FOR FOOD PREPARATION

MODULE 20: HOUSEHOLD FOOD CONSUMPTION – FOR THE LAST 7 DAYS

Respondent MEMID: ____________________

20.0 Firstly, we would like to ask the following four questions before we continue to ask you about your household food consumption.

	(Please enter the MEMID, if decision was jointly please enter both MEMID)	**MEMID**	**MEMID**
1	Who is mainly responsible for the food preparation in the household?		
2	Who is mainly responsible for food purchase in the household?		
3	Who is the main decision maker on food expenditure in the household?		
4	Who is the main decision maker on non-food expenditure in the household?		

Now we would like to ask about food consumption in the past seven days. Indicate how much of the following food items your household consumed, the prices in Ksh and the source of its origin (This is for all food consumed in the household, by all the people listed on demographic table in Module 1. INCLUDE food prepared at home but eaten outside. EXCLUDE meals prepared outside the home).

Ask how many people were present in the last 7 days? Please note down the number of household members in the following table. Please differentiate between female, male and children, as well as household members and visiting members. Fill in NA if a food item was not consumed in the last 7 days.

5	6	7	8	9	10	11	12
Household members				Visiting members			
**Adults**		**Children**		**Adults**		**Children**	
Male	*Female*	*Male*	*Female*	*Male*	*Female*	*Male*	*Female*

19. During the last 7 days did you have any special day (tea guests, chama, burial, wedding etc.), where you cooked more food than usual? (1=Yes, 0=No) __________________

19.1 If yes to question 19, please specify (list number of people (adult/children)): __________________________________ Number of days: ______________________________

Questionnaire number (adda_hhid) ___________________

	14	15	16	17	17a	17b	17c	18
	**Food Items consumed in the past 7 DAYS**	**How much in total did your household consume during the last 7 days?**	**Unit of quantities consumed** *(Use codes above A)*	**Source (*record quantities*)**				**Average price per purchased unit Ksh…**
				Own production	*Purchased*	*Gift*	*Other, specify*	
1	**Staple foods**							
2	Maize green							
3	Maize grain							
4	Maize flour							
5	Yam Tuber							
6	Yam flour							
7	Cassava Tuber							
8	Cassava flour							
9	Orange fleshed sweet potato							
10	Other sweet potato							
11	Irish potato							
12	Irish potato chips							
13	Arrowroots							
14	Sorghum grain							
15	Sorghum Flour							
16	Millet grain							
17	Millet flour							
18	Brown rice							
19	White rice							
20	Wheat grain							
21	Wheat flour brown							
22	Wheat flour white							
23	Cooking banana							
24	**Other staple foods**							
25								
26								
27								
28	**Vegetables**							
29	Amaranth leaves (Emboga)							
30	Black night shade (Rinagu)							
31	Butternut							
32	Cabbage							

	14	15	16	17	17a	17b	17c	18
	**Food Items consumed in the past 7 DAYS**	**How much in total did your household consume during the last 7 days?**	**Unit of quantities consumed** *(Use codes above A)*	**Source (*record quantities*)**				**Average price per purchased unit Ksh…**
				Own production	*Purchased*	*Gift*	*Other, specify*	
33	Carrot							
34	Cow pea leaves							
35	Cucumber							
36	Eggplant							
203	Ginger							
37	Jute mallow (Omotere)							
38	Kales (Sukuma)							
39	Mushrooms							
40	Okra							
41	Onion							
42	Pepper/Capsicum (Hoho)							
204	Chili/Pepper							
43	Pumpkin							
44	Pumpkin leaves (Risosa)							
45	Spider plant (Chinsaga)							
46	Spinach							
47	Stinging nettle (rise)							
48	Sweet potato leaves							
49	Tomato							
50	Vine spinache (Enerema)							
51	**Other vegetables**							
52								
53								
54								
55	**Nuts and Pulses**							
200	KK 15 (fresh+dry)							
56	Beans dry							
57	Beans fresh							
58	Black Bean							
59	Cashew nut							

Code A		3	Teaspoon	6	Kg	9	10 kg bag	12	90 kg bag	16	Piece/Counts	19	1 kg tin	25	Pint (500 ml)	33	Black Paper Bag	77	Others Specify
1	Liter	4	Tablespoon	7	Gram	10	25 kg bag	13	Debe	17	¼ kg tin	21	Bundles	26	Gorogoro	34	Green Paper Bag	78	Others Specify
2	Milliliter	5	Serving spoon	8	5 kg bag	11	50 kg bag	14	Bunch	18	½ kg tin	22	Handful	31	Cob	40	Stick	79	Others Specify

Questionnaire number (adda_hhid) ___________________

	14	15	16	17	17a	17b	17c	18
	**Food Items consumed in the past 7 DAYS**	**How much in total did your household consume during the last 7 days?**	**Unit of quantities consumed** *(Use codes above A)*	**Source (*record quantities)*** *Own production*	*Purchased*	*Gift*	*Other, specify*	**Average price per purchased unit Ksh…**
60	Green grams							
61	Groundnut (boild)							
62	Groundnut (roasted)							
63	Lentils							
64	Peas (incl cowpea (Egesare), pigeon peas, green peas)							
65	Sesame seeds							
66	Soya meat							
67	Soybean							
68	Soybean flour							
69	**Other pulses and nuts**							
70								
71								
72								
73	**Fruits**							
74	Apple							
75	Avocado							
76	Coconut							
77	Guava							
78	Melon							
79	Orange							
80	Passion fruit							
81	Physalis/goose berry							
82	Pineapple							
83	Ripe bananas							
84	Ripe mango							
85	Ripe pawpaw							
86	Sugar cane							
87	**Other fruits**							
88								
89								
90								
91	**Meat and animal Products**							
92	Beef sausage							

	14	15	16	17	17a	17b	17c	18
	**Food Items consumed in the past 7 DAYS**	**How much in total did your household consume during the last 7 days?**	**Unit of quantities consumed** *(Use codes above A)*	**Source (*record quantities)*** *Own production*	*Purchased*	*Gift*	*Other, specify*	**Average price per purchased unit Ksh…**
93	Bush meat (Game meat)							
201	Kuroiler Chicken							
94	Chicken							
95	Chicken sausage							
96	Cow meat							
202	Kuroiler Eggs (pieces)							
97	Eggs (pieces)							
98	Fish							
99	Goat/ Sheep meat							
100	Liver (from any animal)							
101	Offal's (matumbo)							
102	Pork							
103	Sardine (dagaa)							
104	Termites							
105	Turkey (batamzinga)							
106	**Other meats**							
107								
108								
109								
110	**Dairy products**							
111	Cheese							
112	Ice cream							
113	Milk (cow/goat milk)							
114	Powdered milk							
115	Sour milk (mala)							
116	Yoghurt							
117	**Other dairy product**							
118								
119								
120								
121	**Beverages**							
122	Cocoa powder							

Code A																			
1	Liter	3	Teaspoon	6	Kg	9	10 kg bag	12	90 kg bag	16	Piece/Counts	19	1 kg tin	25	Pint (500 ml)	33	Black Paper Bag	77	Others Specify
2	Milliliter	4	Tablespoon	7	Gram	10	25 kg bag	13	Debe	17	¼ kg tin	21	Bundles	26	Gorogoro	34	Green Paper Bag	78	Others Specify
		5	Serving spoon	8	5 kg bag	11	50 kg bag	14	Bunch	18	½ kg tin	22	Handful	31	Cob	40	Stick	79	Others Specify

	14	15	16	17	17a	17b	17c	18
	**Food Items consumed in the past 7 DAYS**	**How much in total did your household consume during the last 7 days?**	**Unit of quantities consumed** *(Use codes above A)*	**Source (*record quantities)***				**Average price per purchased unit Ksh…**
				Own production	*Purchased*	*Gift*	*Other, specify*	
123	Coffee (powder)							
124	Drinking chocolate							
125	Milo powder							
126	Soya powder							
127	Tea (leaves)							
128	**Other beverages**							
129								
130								
131	**Drinks**							
132	Apple juice							
133	Bottled beer							
134	Local beer							
135	Orange juice							
136	Pineapple juice							
137	Other juice (concentrates)							
138	Soft drinks (coke/fanta/etc)							
139	Wine							
140	**Other drinks**							
141	Water (Drinking)							
142								
143	**Condiments and spices**							
144	Salt							
145	Curry (Powder							
146	Ginger (tangawizi; Powder)							
147	Ketchup, Tomato sauce							
148	Black Pepper (Powder)							
149	**Other Condiments and spices**							
150								
151								
152								

	14	15	16	17	17a	17b	17c	18
	**Food Items consumed in the past 7 DAYS**	**How much in total did your household consume during the last 7 days?**	**Unit of quantities consumed** *(Use codes above A)*	**Source (*record quantities)***				**Average price per purchased unit Ksh…**
				Own production	*Purchased*	*Gift*	*Other, specify*	
153	**Snacks (Bought ones, don't account into flour)**							
154	Bread							
155	Biscuit/cookies							
156	Popcorn							
157	Cakes							
158	Mandazi							
159	**Other snacks**							
160								
161								
162	**Sugar and sweets**							
163	Sugar							
164	Chocolate							
165	Honey							
166	Sweets							
167	**Other sugar and sweets**							
168								
169								
170	**Fat and Oil**							
171	Animal fat							
172	Butter							
173	Corn Oil							
174	Groundnut Oil							
175	Margarine							
176	Sunflower Oil							
177	Vegetable Oil							
178	Vegetables Fat							
179	**Other oil**							
180								
181								

Code A		3	Teaspoon	6	Kg	9	10 kg bag	12	90 kg bag	16	Piece/Counts	19	1 kg tin	25	Pint (500 ml)	33	Black Paper Bag	77	Others Specify
1	Liter	4	Tablespoon	7	Gram	10	25 kg bag	13	Debe	17	¼ kg tin	21	Bundles	26	Gorogoro	34	Green Paper Bag	78	Others Specify
2	Milliliter	5	Serving spoon	8	5 kg bag	11	50 kg bag	14	Bunch	18	½ kg tin	22	Handful	31	Cob	40	Stick	79	Others Specify

Questionnaire number (adda_hhid) ___________________

TARGET PERSON: Mother or caretaker of child between the age of six to 59 months

MODULE 21: CHILD QUESTIONNAIRE – ONLY ONE CHILD WILL BE CONSIDERED

Respondent MEMID: _______________

Please only ask this section to households that have children **under the age of 5 years**. Check first and probe if you are not sure! Ask the following questions to the person who is mainly responsible for the child care, e.g. mother, father, grandmother etc.)

If **NO** Child under 5 years is living in the household, please go to the next Module 22. If more than one child is under the age of 5 years, please choose the child of the group member. If not applicable choose child where respondent is available. If there is more than one child in the age range please choose randomly.

1	2	3a	3b	3c	4	5	6
MEMID of child	Name	Date of Birth *(please check with birth card; don't know=88)*			Age in month	Health card present? (No=0, Yes=1)	Which relationship to Child do you have? *(Respondent of this section, insert code below)* **Code A**
		Day	Month	Year			

Code A

1	Father	6	Stepfather/stepmother
2	Mother	7	Cousin
3	Grandmother/grandfather	8	Remote relative
4	Sister/brother	9	Care taker
5	Aunt/uncle	77	Others, specify______

PART 1: CHILD ANTHROPOMETRIC

In this section we would like to take the height, weight and mid-upper arm circumferences of your child. We will do this twice by two different enumerators to make sure to get the right weight and height.

1	1a	2	2a	3	3a	3b
Weight (kg)		Height (cm)		MUAC (measure tape)		
Data	Data	Data	Data	Data	Data	Data

Questionnaire number (adda_hhid) ___________________

PART 2: CHILD HEALTH SECTION

Please ask the parent/caretaker of the child the following questions.

1	Where did you give birth to this child? **A**	
2	Was the child a single or a multiple birth? *Single = 0, Twin = 1, Triple=3, Other, specify=77*	
3	What is the child's birth order e.g. was child born 1st, 2nd, 3rd, etc.?	
4	Please record here, if the child received the following vaccines:<br>*(insert 0 if they did not receive,"1" if they did & have card, and a "2" if the mother says the child received but there is no card), don't know =88*<br>a Measles (at 9 month) c BCG (against tuberculosis) – at birth<br>b Polio (OPV – at birth, 6wk, 10 wk and 14 wk) d DPT/DTaP (diphtheria, pertussis e.g. whooping cough and tetanus) (at 6wk, 10 wk and 14 wk)	
5	Has **<u>the selected</u>** child suffered from any of the following illnesses/symptoms in the *<u>last 14 days</u>*? Indicate for how long ____ days<br>a Diarrhea d Fever g Blindness j Mouth problems<br>b Measles e Fatigue/lethargy h Skin diseases k Eye disease<br>c Anaemia f Respiratory illness i Pneumonia l Others, specify	
6	In the last year (1st Oct 2015 to 30th Sep 2016) did the child receive any of the following nutrition supplements or medical treatments (pills, liquids or supplemented food)?<br>If YES, indicate for how long ___________________ days; if No=0, Don't Know=88<br>a Vitamin A (red/blue) b Iron c Iodine d Zinc (OLS)	
7	Has **[considered child]** ever been breastfed? No=0, Yes=1, Don't Know=88	
8	Was [**considered child]** given anything to drink or eat BEFORE the first breastfeed? No=0, Yes=1, Don't Know=88	
9	Is child still being **breastfed?** No=0, Yes=1, Don't Know=88	
10	If the child is not breastfed anymore, at what age (in month) was breastfeeding stopped? *(please record age in month)*	
11	At what age was [considered child] given **other food** apart from breast milk?<br>*Please verify by asking other household members; please record age in month or if not sure = 88, or if child does not take food yet =99*	
12	What do you think is the recommended period of exclusive breastfeeding? *Define exclusive breastfeeding ,if unsure, fill in 88 –* **Record Number of month**	
13	Which health problems or disease do you think are associated with **not exclusively breastfeeding**? *(Maximum 3 options) B*	**a** **b** **c**

Code A
1 Health facility
2 Home
3 En route to facility
4 Private hospital
6 Outside of home (e.g. in the field)
7 At another home (e.g. relative, neighbor)
8 Government hospital
77 Other, specify__________________
88 Do not Know

Code B
1 Death
2 Low weight for height
3 Low height for age
4 Low weight
5 Stomach ache
6 Delayed achievement of development milestones (smiling, grabbing, etc)
7 Diarrhoea
8 Weakness
9 Low immunity
10 Obesity
77 Other, specify______________
99 None
88 DNK

Questionnaire number (adda_hhid) ____________________

PART 3 Child: Dietary Recall from YESTERDAY (24hr DR) → INDICATE ONLY FOOD ITEMS

CHILD MEMID: ______________________________________

Now I would like to ask you about everything (including snacks) that (CHILD) ate yesterday during the day or night, whether at home or outside of the home.*Continue through the day,* ***until respondent indicates child went to sleep*** *until the next day. If respondent mentions a mixed dish like a porridge, relish or stew, ask about all ingredients that went into the dish, including added oil, sugar or condiments.* ***Probe for drinks & fruits.***

1	Which day of the week does this record represent? **A**		
2	Is this a typical day? YES =1, NO=0		13. If NO, please specify:

3	4	5	6	7	8	9	10	11	12
**Main Dish**	**Quantity**	**Unit -D**	How was the **dish** prepared-**C**	**Ingredients**	**Quantity**	**Unit -D**	Source of Ingredients **B**	No of People ate from the Main Dish	Where was the food consumed? - **E**
**Breakfast**									
**Snack**									
**Lunch**									
**Snack**									
**Dinner**									
**Snack**									

**Code A**			
1	Monday	4	Thursday
2	Tuesday	5	Friday
3	Wednesday	6	Saturday
		7	Sunday

**Code B**		4	Rain Water
1	Own production	5	Spring Water
2	Purchased	6	Well
3	Gift	7	Borehole
		77	Other, Specify

**Code C**		5	Cooked
1	Raw	6	Fried
2	Dried	7	Roasted
3	Boiled	8	Processed
4	Steamed	77	Other, specify

**Code E**		5	Market
1	Home	6	School
2	Hotel	7	Ceremony
3	Friend	77	Other, specify____
4	Work		

**Code D**		3	Tablespoon	6	Pinch	9	Serving spoon	13	Glass	21	Slice	25	Bowl	77	Other, specify____
1	Small cup	4	Grams	7	Ml	10	Piece	16	Bundle	23	Cobs	26	Handful		
2	Teaspoon	5	Liter	8	Pint (500ml)	12	Cup	17	Kg	24	Plates	40	Stick		

Questionnaire number (adda_hhid) ____________________

MODULE 22: INDIVIDUAL PART

Respondent MEMID _______________

Name: __________________________

MODULE 22/1- FIRST INDIVIDUAL QUESTIONNAIRE (1)

This section will be answered by one chosen individual of the household, e.g. individual which is mentioned in the information sheet.

This module will be asked at two different days. Additionally, we would like to measure the respondent (weight, height, hip and waist). Please tell the respondent the date of the second visit.
Please make sure that the same person on both days is answering the questionnaire.

**PART 3: INDIVIDUAL 1 - ANTHROPOMETRIC**						
In this section we would like to take the height and weight of you. We will do this twice by two different enumerators to make sure to get the right weight and height.						
1	2	2a	3	3a	4	5
Type of clothe (light=0, heavy=1)	Weight (kg)		Height (cm)		Waist	Hip
	Data	Data	Data	Data	cm	cm

Questionnaire number (adda_hhid) ____________________

PART 1 First Individual Dietary Recall for YESTERDAY (1) (24hr DR) → Record Day______________________

Respondent MEMID: _______________

*Now we would like to know, which food and drinks and how much of it did you alone (not your household) consume yesterday. Please indicate food item, preparation and sourc. Write down all the foods, **drinks** and **fruits** mentioned. When mixed dishes are mentioned, ask for the list of ingredients. When the respondents has finished, probe for the meals and snacks not mentioned.*

1	Which day of the week does this record represent? **A**								
2	Is this a typical day? YES =1, NO=0				13. If NO, please specify:				
3	4	5	6	7	8	9	10	11	12
**Main Dish**	**Quantity**	**Unit-D**	How was the **dish** prepared-**C**	**Ingredients**	**Quantity**	**Unit-D**	Source of Ingredients **B**	No of People ate from the Main Dish	Where was the food consumed? - **E**
**Breakfast**									
**Snack**									
**Lunch**									
**Snack**									
**Dinner**									
**Snack**									

				**Code B**		4	Rain Water	**Code C**		5	Cooked	**Code E**		5	Market
**Code A**		4	Thursday	1	Own production	5	Spring Water	1	Raw	6	Fried	1	Home	6	School
1	Monday	5	Friday	2	Purchased	6	Well	2	Dried	7	Roasted	2	Hotel	7	Ceremony
2	Tuesday	6	Saturday	3	Gift	7	Borehole	3	Boiled	8	Processed	3	Friend	77	Other, specify____
3	Wednesday	7	Sunday			77	Other, Specify	4	Steamed	77	Other, specify	4	Work		
**Code D**		3	Tablespoon	6	Pinch	9	Serving spoon	13	Glass	21	Slice	25	Bowl	77	Other, specify____
1	Small cup	4	Grams	7	Ml	10	Piece	16	Bundle	23	Cobs	26	Handful		
2	Teaspoon	5	Liter	8	Pint (500ml)	12	Cup	17	Kg	24	Plates	40	Stick		

Questionnaire number (adda_hhid) ____________________

Second visit (Day 2) **PART 2: First Individual: Dietary Recall for YESTERDAY (2) (24hr DR)**

Record Day__________________ **Respondent MEMID: _______________**

Now we would like to know, which food and drinks and how much of it did you alone (not your household) consume yesterday. Please indicate food item, preparation and sourc. Write down all the foods, ***drinks*** *and* ***fruits*** *mentioned. When mixed dishes are mentioned, ask for the list of ingredients. When the respondents has finished, probe for the meals and snacks not mentioned.*

1	Which day of the week does this record represent? **A**		
2	Is this a typical day? YES =1, NO=0		13. If NO, please specify:

3	4	5	6	7	8	9	10	11	12
**Main Dish**	**Quantity**	**Unit -D**	How was the **dish** prepared-**C**	**Ingredients**	**Quantity**	**Unit -D**	Source of Ingredients **B**	No of People ate from the Main Dish	Where was the food consumed? - **E**
**Breakfast**									
**Snack**									
**Lunch**									
**Snack**									
**Dinner**									
**Snack**									

**Code A**			
1	Monday	4	Thursday
2	Tuesday	5	Friday
3	Wednesday	6	Saturday
		7	Sunday

**Code B**			
1	Own production	4	Rain Water
2	Purchased	5	Spring Water
3	Gift	6	Well
		7	Borehole
		77	Other, Specify

**Code C**			
1	Raw	5	Cooked
2	Dried	6	Fried
3	Boiled	7	Roasted
4	Steamed	8	Processed
		77	Other, specify

**Code E**			
1	Home	5	Market
2	Hotel	6	School
3	Friend	7	Ceremony
4	Work	77	Other, specify____

**Code D**																	
1	Small cup	3	Tablespoon	6	Pinch	9	Serving spoon	13	Glass	21	Slice	25	Bowl	77	Other, specify____
2	Teaspoon	4	Grams	7	Ml	10	Piece	16	Bundle	23	Cobs	26	Handful		
		5	Liter	8	Pint (500ml)	12	Cup	17	Kg	24	Plates	40	Stick		

Code A

1	very good	2	Good	3	not good/not poor	4	a little poor	5	very poor	88	DNK

Code B

1	Asthma	3	Cardiovascular disease	4	Diabetes	5	Hypertension	77	Other, specify_
88	DNK	99	None						

Code C

1	Bad teeth (ache)	5	Eye problems	9	Headache	13	Malaria	17	Stomach ache	21	Vomiting
2	Cholera	6	Fainting	10	Hepatitis	14	Measles	18	Tetanus	22	Weakness
3	Diarrhea	7	Fever	11	High cholesterol	15	Pneumonia	19	Tiredness/Fatigue	77	Other, specify__
4	Ear/throat problem	8	Flu/Cold	12	Intestinal worms	16	Skin Problem	20	Tuberculosis	99	None

Code D

1	Fatigue, tiredness	3	Weak immune system	5	Reduction in intelligence	7	Soreness of the moth	77	Other, specify____
2	Low concentration	4	Shortage of blood	6	Small stature	8	Unusual quantity of hair loss	88	DNK

Code E

1	Green leafy vegetables	3	Soybean/chick peas/ lentils	5	Fish	7	Liver	77	Other, specify
2	Peas	4	Meat	6	orange/red coloured fruits & vegetables (eg., OFSP, tomatoes)			88	DNK

Code F

1	leading to eye blindness	2	Night blindness	3	Measles	4	Diarrhea	5	Worms
77	Other, specify_	88	DNK						

Code G

1	Green leafy vegetables	3	Milk & milk products	5	Eggs	77	Other, specify_
2	Orange vegetables & fruits	4	Palm Oil	6	Offal's	88	DNK

Code H

1	Iodine	3	Vitamin A	5	Zinc	77	Other specify	99	None
2	Iron	4	Vitamin C			88	DNK		

Code J

1	**Beans**	3	Cheese	5	Dairy products	7	Fish	9	Sardines	77	Other, specify
2	Beef	4	Chicken	6	Eggs	8	Milk	10	Yoghurt	88	DNK

Code K -

1	Chapatti	3	Cassava	5	Crips	7	Plantain	9	Ugali	88	DNK
2	Bread	4	Yam	6	Potato	8	Rice	77	Other, specify		

Code L

1	Butter	3	Lard	5	Oil	77	Other, specify
2	Groundnuts	4	Kimbo	6	Palm Oil	88	DNK

Code M

1	Radio	4	Doctor	7	Health centre	10	School	77	Other Specify
2	TV	5	**Nutrition education (specify)**	8	Internet	11	Community health worker	88	DNK
3	**NGO (specify:_____)**	6	Newspaper	9	Friends/Relatives	12	Church		

Code N

1	Hypertension	3	Diabetes	5	High cholesterol
2	Cardiovascular diseases	4	Cancer	77	Others, specify

Code O

1	Respondent	3	House help	5	Sister	7	Daughter	9	Respindent + Spouse	77	Other specify:
2	Spouse	4	Brother	6	Grandparents	8	Son	10	Whole family	99	N/A

Questionnaire number (adda_hhid) ____________________

PART 2 First Individual: INDIVIDUAL KNOWLEDGE QUESTIONS - second visit (Day 2)

Respondent MEMID: _______________

1	During the last 4 weeks how would you rate your health? **A**					
2	Do you suffer from any of the following diseases __________________ **[Read out Code B]**					
3	Have you suffered from any diseases or symptoms during the last 30 days?<br>*Allow up to four answers* **C**	a	b	c	d	
4	Can you name anything that helps prevent you and other family members from getting diarrhea? Yes=1, No=0,<br>***If No, skip to question 5*_______.**<br>If yes, let respondent specify and tick the ones that apply underneath – *do not read out the list, probe for further responses – more than one answer possible*<br>a Washing hands; c Exclusive breast feeding; e Other specify<br>b Use latrine/ bury faces; d Protect food & water supplies with cover					
5	Which diseases or problems are caused by deficiency in iron?*(allow up to3 answers- **Do not prompt**)* **D**	a	b	**c**		
6	Do you know which foods have ample amounts of iron? *(allow up to3 answers – **Do not prompt**)* **E**	a	b	**c**		
7	Which diseases or problems are caused by deficiency in vitamin A? *(allow up to3 answers – **Do not prompt**)* **F**	a	b	**c**		
8	Do you know which foods have ample amounts of vitamin A? *(allow up to3 answers – **Do not prompt**)* **G**	a	b	**c**		
10	Did [NAME] receive any nutritious supplements in the last year (Oct 15 – Sept 16)?<br>*Multiple answer possible, allow up to 3 answers* **H**	a	b	c		
11	Who do you think is the most informed person within your household concerning nutrition information? **ENTER MEMID**					
12	Who do you think is the most informed person within your household concerning agricultural information? **ENTER MEMID**					
13	Have you heard of the following macronutrients:<br>*No=0, Yes=1*; *If no, please go to question 24*	a Protein	b Carbohydrates	c Fat		
14	If yes, to question 20, please name which food items can supply those macronutrients:<br>*(allow up to three answers)*			a	b	c
		Protein	**J**			
15		Carbohydrates	**K**			
16		Fat	**L**			
19	During the last year (Oct. 2015 - Sept 2016), have you noticed or received information/training about healthy eating or healthy diets? *No=0, Yes=1 (If NO, skip to next M23)*					
20	If yes, where did you find, see or get this information on healthy eating/diet? **M**					
21	Are you aware of any health problems or diseases that are associated with excess weight? *Yes = 1 No = 0 Don't know = 88*					
22	Which diseases do you think are associated with excess weight? *(Allow up to three responses. Rank according to likelihood)* **N**	1	2	3		

Modul 23/1: Decision making

		**Code O**	Fill in MEMID
1	Who decides how the household finances should be allocated?		
2	Who manages the household finances and implements the decision?		
3	Who makes the decision **which** food is purchased in the household?		
4	If not the respondent, please ask if the respondent would prefer other food to be purchased. YES=1, NO=0		
5	Who makes the decision **where** food is purchased in the household?		
6	If not the respondent, please ask if the respondent would prefer that the food would be purchased somewhere else? YES=1, NO=0		
7	Who decides which and how much food is consumed from your own produce?		
8	Who is responsible to purchases food on a daily basis?		
9	Who makes the decision which food is prepared in the household?		
10	Who is responsible for daily food preparation in the household?		
11	Who makes decisions on expenditure on cheap household assets?		
12	Who males decisions on expenditure on expensive household assets?		
13	Who makes decisions on medical expenditures?		
14	Who makes decisions on education?		
15	Who decides to participate in agricultural training sessions?		

Questionnaire number (adda_hhid) ___________________

Module 22: Individual Part

Respondent MEMID _______________

Name: ___________________________

MODULE 22/2- SECOND INDIVIDUAL QUESTIONNAIRE (2)

This section will be answered by one chosen individual of the household,e.g the second individual on the information sheet given.

This module will be asked at two different days. At the first visit one 24-hour dietary recall will be asked. On the second visit a second 24 hour dietary recall and nutrition/health knowledge questions will be asked. Additionally, we would like to measure the respondent (weight, height, hip and waist). Please make an appointment with the respondent to come back after at least two days. Please make sure that the same person on both days is answering the questionnaire.

**PART 3: INDIVIDUAL 2 ANTHROPOMETRIC**						
In this section we would like to take the height, weight of you. We will do this twice by two different enumerators to make sure to get the right weight and height.						
1	2	2a	3	3a	4	5
Type of clothe (light=0, heavy=1)	Weight (kg)		Height (cm)		Waist	Hip
	Data	Data	Data	Data	cm	cm

Questionnaire number (adda_hhid) ___________________

PART 1 Second Individual: Dietary Recall for YESTERDAY (1) (24hr DR) → Record Day

Respondent MEMID:_______________

*Now we would like to know, which food and drinks and how much of it did you alone (not your household) consume yesterday. Please indicate food item, preparation and sourc. Write down all the foods, **drinks** and **fruits** mentioned. When mixed dishes are mentioned, ask for the list of ingredients. When the respondents has finished, probe for the meals and snacks not mentioned.*

1	Which day of the week does this record represent? **A**								
2	Is this a typical day? YES =1, NO=0				13. If NO, please specify:				
3	4	5	6	7	8	9	10	11	12
**Main Dish**	**Quantity**	**Unit -D**	How was the **dish** prepared-**C**	**Ingredients**	**Quantity**	**Unit -D**	Source of Ingredients **B**	Nb of People eaten from Main Dish	Where was the food consumed? - **E**
**Breakfast**									
**Snack**									
**Lunch**									
**Snack**									
**Dinner**									
**Snack**									

**Code A**			
1	Monday	4	Thursday
2	Tuesday	5	Friday
3	Wednesday	6	Saturday
		7	Sunday

**Code B**			
1	Own production	4	Rain Water
2	Purchased	5	Spring Water
3	Gift	6	Well
		7	Borehole
		77	Other, Specify

**Code C**			
1	Raw	5	Cooked
2	Dried	6	Fried
3	Boiled	7	Roasted
4	Steamed	8	Processed
		77	Other, specify

**Code E**			
1	Home	5	Market
2	Hotel	6	School
3	Friend	7	Ceremony
4	Work	77	Other, specify____

**Code D**															
1	Small cup	3	Tablespoon	6	Pinch	9	Serving spoon	13	Glass	21	Slice	25	Bowl	77	Other, specify____
2	Teaspoon	4	Grams	7	Ml	10	Piece	16	Bundle	23	Cobs	26	Handful		
		5	Liter	8	Pint (500ml)	12	Cup	17	Kg	24	Plates	40	Stick		

Questionnaire number (adda_hhid) ____________________

Second visit (Day 2) **PART 2Second Individual: Dietary Recall for YESTERDAY (2) (24hr DR)**

Record Day_________________ **Respondent MEMID:_______________**

Now we would like to know, which food and drinks and how much of it did you alone (not your household) consume yesterday. Please indicate food item, preparation and sourc. Write down all the foods, ***drinks*** *and* ***fruits*** *mentioned. When mixed dishes are mentioned, ask for the list of ingredients. When the respondents has finished, probe for the meals and snacks not mentioned.*

1	Which day of the week does this record represent? **A**								
2	Is this a typical day? YES =1, NO=0				13. If NO, please specify:				
3	4	5	6	7	8	9	10	11	12
**Main Dish**	**Quantity**	**Unit -D**	How was the **dish** prepared-**C**	**Ingredients**	**Quantity**	**Unit -D**	Source of Ingredients **B**	Nb of People eaten from Main Dish	Where was the food consumed? -**E**
**Breakfast**									
**Snack**									
**Lunch**									
**Snack**									
**Dinner**									
**Snack**									

**Code A**		4	Thursday
1	Monday	5	Friday
2	Tuesday	6	Saturday
3	Wednesday	7	Sunday

**Code B**		4	Rain Water
1	Own production	5	Spring Water
2	Purchased	6	Well
3	Gift	7	Borehole
		77	Other, Specify

**Code C**		5	Cooked
1	Raw	6	Fried
2	Dried	7	Roasted
3	Boiled	8	Processed
4	Steamed	77	Other, specify

**Code E**		5	Market
1	Home	6	School
2	Hotel	7	Ceremony
3	Friend	77	Other, specify____
4	Work		

**Code D**		3	Tablespoon	6	Pinch	9	Serving spoon	13	Glass	21	Slice	25	Bowl	77	Other, specify____
1	Small cup	4	Grams	7	Ml	10	Piece	16	Bundle	23	Cobs	26	Handful		
2	Teaspoon	5	Liter	8	Pint (500ml)	12	Cup	17	Kg	24	Plates	40	Stick		

Code A

1	very good	2	Good	3	not good/not poor	4	a little poor	5	very poor	88	DNK

Code B

1	Asthma	3	Cardiovascular disease	4	Diabetes	5	Hypertension	77	Other, specify_
88	DNK	99	None						

Code C

1	Bad teeth (ache)	5	Eye problems	9	Headache	13	Malaria	17	Stomach ache	21	Vomiting
2	Cholera	6	Fainting	10	Hepatitis	14	Measles	18	Tetanus	22	Weakness
3	Diarrhea	7	Fever	11	High cholesterol	15	Pneumonia	19	Tiredness/Fatigue	77	Other, specify__
4	Ear/throat problem	8	Flu/Cold	12	Intestinal worms	16	Skin Problem	20	Tuberculosis	99	None

Code D

1	Fatigue, tiredness	3	Weak immune system	5	Reduction in intelligence	7	Soreness of the moth	77	Other, specify____
2	Low concentration	4	Shortage of blood	6	Small stature	8	Unusual quantity of hair loss	88	DNK

Code E

1	Green leafy vegetables	3	Soybean/chick peas/ lentils	5	Fish	7	Liver	77	Other, specify
2	Peas	4	Meat	6	orange/red coloured fruits & vegetables (eg., OFSP, tomatoes)			88	DNK

Code F

1	leading to eye blindness	2	Night blindness	3	Measles	4	Diarrhea	5	Worms
77	Other, specify_	88	DNK						

Code G

1	Green leafy vegetables	3	Milk & milk products	5	Eggs	77	Other, specify_
2	Orange vegetables & fruits	4	Palm Oil	6	Offal's	88	DNK

Code H

1	Iodine	3	Vitamin A	5	Zinc	77	Other specify	99	None
2	Iron	4	Vitamin C			88	DNK		

Code J

1	**Beans**	3	Cheese	5	Dairy products	7	Fish	9	Sardines	77	Other, specify
2	Beef	4	Chicken	6	Eggs	8	Milk	10	Yoghurt	88	DNK

Code K -

1	Chapatti	3	Cassava	5	Crips	7	Plantain	9	Ugali	88	DNK
2	Bread	4	Yam	6	Potato	8	Rice	77	Other, specify		

Code L

1	Butter	3	Lard	5	Oil	77	Other, specify
2	Groundnuts	4	Kimbo	6	Palm Oil	88	DNK

Code M

1	Radio	4	Doctor	7	Health centre	10	School	77	Other Specify
2	TV	5	**Nutrition education (specify)**	8	Internet	11	Community health worker	88	DNK
3	**NGO (specify:_____)**	6	Newspaper	9	Friends/Relatives	12	Church		

Code N

1	Hypertension	3	Diabetes	5	High cholesterol
2	Cardiovascular diseases	4	Cancer	77	Others, specify

Code O

1	Respondent	3	House help	5	Sister	7	Daughter	9	Respindent + Spouse	77	Other specify:
2	Spouse	4	Brother	6	Grandparents	8	Son	10	Whole family	99	N/A

Questionnaire number (adda_hhid) ____________________

PART 2 Second Individual: INDIVIDUAL KNOWLEDGE QUESTIONS - second visit (Day 2)

Respondent MEMID: ______________

1	During the last 4 weeks how would you rate your health? **A**				
2	Do you suffer from any of the following diseases __________________ **[Read out Code B]**				
3	Have you suffered from any diseases or symptoms during the last 30 days? *Allow up to four answers* **C**	a	b	c	d
4	Can you name anything that helps prevent you and other family members from getting diarrhea? Yes=1, No=0, ***If No, skip to question 5*_______.** If yes, let respondent specify and tick the ones that apply underneath – *do not read out the list, probe for further responses – more than one answer possible*<br>a Washing hands; c Exclusive breast feeding; e Other specify<br>b Use latrine/ bury faces; d Protect food & water supplies with cover				
5	Which diseases or problems are caused by deficiency in iron?*(allow up to3 answers- **Do not prompt**)* **D**	a	b	**c**	
6	Do you know which foods have ample amounts of iron? *(allow up to3 answers – **Do not prompt**)* **E**	a	b	**c**	
7	Which diseases or problems are caused by deficiency in vitamin A? *(allow up to3 answers – **Do not prompt**)* **F**	a	b	**c**	
8	Do you know which foods have ample amounts of vitamin A? *(allow up to3 answers – **Do not prompt**)* **G**	a	b	**c**	
10	Did [NAME] receive any nutritious supplements in the last year (Oct 15 – Sept 16)? *Multiple answer possible, allow up to 3 answers* **H**	a	b	c	
11	Who do you think is the most informed person within your household concerning nutrition information? **ENTER MEMID**				
12	Who do you think is the most informed person within your household concerning agricultural information? **ENTER MEMID**				
13	Have you heard of the following macronutrients: *No=0, Yes=1*; *If no, please go to question 24*	a Protein	b Carbohydrates	c Fat	

				a	b	c
14	If yes, to question 20, please name which food items can supply those macronutrients: *(allow up to three answers)*	Protein	**J**			
15		Carbohydrates	**K**			
16		Fat	**L**			

19	During the last year (Oct. 2015 - Sept 2016), have you noticed or received information/training about healthy eating or healthy diets? *No=0, Yes=1 (If NO, skip to next M23)*			
20	If yes, where did you find, see or get this information on healthy eating/diet? **M**			
21	Are you aware of any health problems or diseases that are associated with excess weight? *Yes = 1 No = 0 Don't know = 88*			
22	Which diseases do you think are associated with excess weight? *(Allow up to three responses. Rank according to likelihood)* **N**	1	2	3

Modul 23/1: Decision making

		**Code O**	Fill in MEMID
1	Who decides how the household finances should be allocated?		
2	Who manages the household finances and implements the decision?		
3	Who makes the decision **which** food is purchased in the household?		
4	If not the respondent, please ask if the respondent would prefer other food to be purchased. *YES=1, NO=0*		
5	Who makes the decision **where** food is purchased in the household?		
6	If not the respondent, please ask if the respondent would prefer that the food would be purchased somewhere else? *YES=1, NO=0*		
7	Who decides which and how much food is consumed from your own produce?		
8	Who is responsible to purchases food on a daily basis?		
9	Who makes the decision which food is prepared in the household?		
10	Who is responsible for daily food preparation in the household?		
11	Who makes decisions on expenditure on cheapl household assets?		
12	Who males decision on expenditure on expensive household assets?		
13	Who makes decisions on medical expenditures?		
14	Who makes decisions on education?		
15	Who decides to participate in agricultural training sessions?		
