# Supplementary material for: Various forms of double burden of malnutrition problems exist in rural Kenya
Source: BMC Public Health. 2019 Nov 21;19:1543. doi: 10.1186/s12889-019-7882-y (PMC6873738; doi:10.1186/s12889-019-7882-y)
Supplement: Supplementary file 2 — Additional file 2: Table S2. Estimated average requirements (EAR) for micronutrients [file 12889_2019_7882_MOESM2_ESM.pdf]

Additional Material A2

Table A2 Estimated average requirements (EAR) for micronutrients

Gender	Age	Iron (mg/d)	Zinc (mg/d)	Vitamin A (μg/d)
Male	19-30y	7.7	9.4	630
	31-50y	6.0	9.4	625
	51-70y	6.0	9.4	625
	>70y	6.0	9.4	625
Female	14-18y	7.9	7.3	485
	19-30y	8.1	6.8	500
	31-50y	8.1	6.8	500
	51-70y	5.0	6.8	500
	>70y	5.0	6.8	500
Child	6-12 m	6.9	2.5	210
	1-3y	3.0	2.5	210
	4-8y	4.1	4.0	275

y, year; m, month
Source: IOM (2006).
