# Supplementary material for: Various forms of double burden of malnutrition problems exist in rural Kenya
Source: BMC Public Health. 2019 Nov 21;19:1543. doi: 10.1186/s12889-019-7882-y (PMC6873738; doi:10.1186/s12889-019-7882-y)
Supplement: Supplementary file 3 — Additional file 3: Table S3. Nutritional status of adults and children in the sample [file 12889_2019_7882_MOESM3_ESM.pdf]

Additional Material A3

Table A3 Nutritional status of adults and children in the sample

**Adults**	Total (n=874)		Female (n=558)		Male (n=316)	
	n	%	n	%	n	%
Underweight (BMI < 18.5)	43	4.9	21	3.8	22	7.0
Normal weight (BMI 18.5 – 24.9)	426	48.7	239	42.8	187	59.2
Overweight/obese (BMI ≥ 25.0)	405	46.3	298	53.4	107	33.9
Overweight (BMI ≥ 25.0 and < 30.0)	265	30.3	182	32.6	83	26.3
Obese (BMI ≥ 30.0)	140	16.0	116	20.8	24	7.6
**Children**	Total (n=184)		Female (n=89)		Male (n=95)	
Severe underweight (BAZ < -3 SD)	1	0.5	0		1	0.5
Severe underweight (WAZ < -3 SD)	2	1.1	0		2	2.1
Severe stunting (HAZ < -3 SD)	16	8.7	7	7.4	9	10.1
Severe wasting (WHZ < - 3SD)	1	0.5	0		1	0.5
Underweight (BAZ < -2 SD)	2	1.1	1	1.1	1	1.1
Underweight (WAZ < -2 SD)	12	6.5	6	6.7	6	6.3
Stunting (HAZ < -2 SD)	42	22.8	19	21.4	23	24.2
Wasting (WHZ < - 2 SD)	2	1.1	1	1.1	1	1.1
Mild Underweight (BAZ < -1 SD)	13	7.1	6	6.7	7	7.4
Mild Underweight (WAZ < -1 DS)	51	27.7	24	27.0	27	28.4
Mild stunting (HAZ < -1 DS)	93	50.5	39	43.8	54	65.8
Mild wasting (WHZ < -1 DS)	14	7.6	5	5.6	9	9.5
At risk of overweight (BAZ > +1 SD)	51	27.7	25	28.1	26	27.4
At risk of overweight (WHZ > +1 SD)	41	22.3	21	23.6	20	21.1
Overweight (BAZ > +2 SD)	6	3.3	1	1.1	5	5.3
Overweight (WHZ > +2 SD)	6	3.3	1	1.1	5	5.3

n, sample size; BMI, body mass index; BAZ, body mass index-for-age Z-score; SD, standard deviation; WAZ, weight-for-height Z-score; HAZ, height-for-age Z-score; WHZ, weight-for-height Z-score
