# Supplementary material for: Various forms of double burden of malnutrition problems exist in rural Kenya
Source: BMC Public Health. 2019 Nov 21;19:1543. doi: 10.1186/s12889-019-7882-y (PMC6873738; doi:10.1186/s12889-019-7882-y)
Supplement: Supplementary file 4 — Additional file 4: Table S4. Prevalence of micronutrient deficiencies in adults and children [file 12889_2019_7882_MOESM4_ESM.pdf]

Additional Material A4

Table A4 Prevalence of micronutrient deficiencies in adults and children

	Micronutrient deficiency		VA deficiency		Zinc deficiency		Iron deficiency	
	n	%	n	%	n	%	n	%
**Adults**								
Total (n=874)	361	41.3	97	11.1	313	35.8	16	1.8
Female (n=558)	185	33.2	50	9.0	158	28.3	14	2.5
Male (n=316)	176	55.7	47	14.9	155	49.1	2	0.6
**Children**								
Total (n=184)	56	30.4	15	8.2	47	25.5	17	9.3
Female (n=89)	28	31.5	10	11.2	19	21.4	5	5.8
Male (n=95)	28	29.5	5	5.3	28	29.5	12	12.6

VA, vitamin A; n, sample size
