# Supplementary material for: Various forms of double burden of malnutrition problems exist in rural Kenya
Source: BMC Public Health. 2019 Nov 21;19:1543. doi: 10.1186/s12889-019-7882-y (PMC6873738; doi:10.1186/s12889-019-7882-y)
Supplement: Supplementary file 5 — Additional file 5: Table S5. Characteristics of adults, female and male, with and without individual-level DB [file 12889_2019_7882_MOESM5_ESM.pdf]

Additional Material A5

Table A5 Characteristics of adults, female and male, with and without individual-level DB

	Total adults			Female adults		Male adults	
	Total (n=874)	Without DB 1 (n=710)	With DB 1 (n=164)	Without DB 1 (n=459)	With DB 1 (n=99)	Without DB 1 (n=251)	With DB 1 (n=65)
Individual age (years)	46.2 (12.9)	45.6 (13.0)	48.5*** (12.56)	43.5 (12.8)	44.4 (11.8)	49.4 (12.5)	54.8*** (11.1)
Individual education (years)	8.7 (3.5)	8.7 (3.6)	8.5 (3.4)	8.4 (3.6)	8.0 (3.1)	9.3 (3.4)	9.4 (3.5)
Kisii county (1/0)	0.7 (0.5)	0.7 (0.5)	0.8*** (0.4)	0.7 (0.5)	0.7 (0.4)	0.7 (0.5)	0.9*** (0.3)
Male household head (1/0)	0.8 (0.4)	0.8 (0.4)	0.8 (0.4)	0.8 (0.4)	0.7 (0.4)	1.0 (0.1)	1.0 (0.0)
Age of household head (years)	50.6 (12.3)	50.1 (12.6)	52.7** (11.0)	50.2 (12.5)	51.1 (10.8)	50.0 (12.8)	55.0*** (11.1)
Farming occupation of head (1/0)	0.6 (0.5)	0.6 (0.5)	0.6 (0.5)	0.6 (0.5)	0.7 (0.5)	0.7 (0.5)	0.7 (0.5)
Education of head (years)	9.1 (3.9)	9.0 (4.0)	9.1 (3.6)	8.9 (4.3)	9.0 (3.6)	9.3 (3.4)	9.4 (3.5)
Number adults (count)	3.5 (1.5)	3.4 (1.5)	3.7** (1.5)	3.3 (1.5)	3.6* (1.5)	3.5 (1.5)	3.8* (1.5)
Number children (count)	1.6 (1.3)	1.7 (1.3)	1.5* (1.4)	1.7 (1.3)	1.6 (1.4)	1.6 (1.3)	1.3 (1.3)
Income per capita in PPP $/year	4164.2 (12911.8)	4256.3 (14169.7)	3767.0 (4457.0)	4414.0 (17279.2)	3241.7 (3491.7)	3971.2 (4941.2)	4571.4 (5559.5)
Poverty rate (1/0)	0.2 (0.4)	0.2 (0.4)	0.2 (0.4)	0.2 (0.4)	0.2 (0.4)	0.1 (0.3)	0.1 (0.3)
Farm size (acres)	1.3 (1.2)	1.2 (1.1)	1.5** (1.5)	1.2 (1.0)	1.2 (1.0)	1.3 (1.2)	2.0*** (1.9)

DB, double burden of malnutrition; DB 1, adult is overweight/obese and micronutrient-deficient; n, sample size

Mean values are shown with standard deviations in parentheses. Differences in mean values are tested for significance: *p < .1, **p < .05, ***p < .01.
