# Supplementary material for: Various forms of double burden of malnutrition problems exist in rural Kenya
Source: BMC Public Health. 2019 Nov 21;19:1543. doi: 10.1186/s12889-019-7882-y (PMC6873738; doi:10.1186/s12889-019-7882-y)
Supplement: Supplementary file 6 — Additional file 6: Table S6. Characteristics of children with and without individual-level DB [file 12889_2019_7882_MOESM6_ESM.pdf]

Additional Material A6

Table A6 Characteristics of children with and without individual-level DB

	All children									Female Children					Male Children							
	Total (n=184)	Without DB 2 (n=182)	With DB2 (n=2)	Without DB 2.1 (n=183)	With DB2.1 (n=1)	Without DB 3 (n=182)	With DB 3 (n=2)	Without DB 3.1 (n=182)	With DB 3.1 (n=2)	Without DB 2 (n=188)	With DB2 (n=1)	Without DB 2.1 (n=89)	Without DB 3 (n=89)	Without DB 3.1 (n=89)	Without DB 2 (n=94)	With DB2 (n=1)	Without DB 2.1 (n=94)	With DB2.1 (n=1)	Without DB 3 (n=93)	With DB 3 (n=2)	Without DB 3.1 (n=93)	With DB 3.1 (n=2)
Individual age	35.5	35.6	22.5	35.3	59.0	35.5	34.5	35.5	34.5	34.3	15.0	34.1	34.1	34.1	36.8	30.0	36.5	59.0	36.8	34.5	36.8	34.5
(months)	(12.7)	(12.7)	(10.6)	(12.6)	(.)	(12.8)	(3.5)	(12.8)	(3.5)	(12.8)	(.)	(12.9)	(12.9)	(12.9)	(12.5)	(.)	(12.3)	(.)	(12.6)	(3.5)	(12.6)	(3.5)
Breastfed (1/0)	1.0	1.0	1.0	1.0	1.0	1.0	1.0	1.0	1.0	1.0	1.0	1.0	1.0	1.0	1.0	1.0	1.0	1.0	1.0	1.0	1.0	1.0
	(0.2)	(0.2)	(0.0)	(0.2)	(.)	(0.2)	(0.0)	(0.2)	(0.0)	(0.2)	(.)	(0.2)	(0.2)	(0.2)	(0.2)	(.)	(0.2)	(.)	(0.2)	(0.0)	(0.2)	(0.0)
Kisii county (1/0)	0.7	0.7	1.0	0.7	1.0	0.7	0.5	0.7	0.5	0.6	1.0	0.6	0.6	0.6	0.8	1.0	0.8	1.0	0.8	0.5	0.8	0.5
	(0.5)	(0.5)	(0.0)	(0.5)	(.)	(0.5)	(0.7)	(0.5)	(0.7)	(0.5)	(.)	(0.5)	(0.5)	(0.5)	(0.4)	(.)	(0.4)	(.)	(0.4)	(0.7)	(0.4)	(0.7)
Male household head	0.8	0.8	1.0	0.8	0.0	0.8	0.5	0.8	0.5	0.8	1.0	0.8	0.8	0.8	0.8	1.0	0.8	0.0	0.8	0.5	0.8	0.5
(1/0)	(0.4)	(0.4)	(0.0)	(0.4)	(.)	(0.4)	(0.7)	(0.4)	(0.7)	(0.4)	(.)	(0.4)	(0.4)	(0.4)	(0.4)	(.)	(0.4)	(.)	(0.4)	(0.7)	(0.4)	(0.7)
Age of household	46.5	46.4	53.5	46.4	66.0	46.3	62.0*	46.3	62.0*	46.3	51.0	46.3	46.3	46.3	46.6	56.0	46.5	66.0	46.3	62.0**	46.3	62.0**
head (years)	(11.7)	(11.7)	(3.5)	(11.6)	(.)	(11.6)	(15.6)	(11.6)	(15.6)	(12.7)	(.)	(12.6)	(12.6)	(12.6)	(10.8)	(.)	(10.7)	(.)	(10.5)	(15.6)	(10.5)	(15.6)
Farming occupation	0.6	0.6	0.5	0.6	1.0	0.6	0.5	0.6	0.5	0.6	0.0	0.6	0.6	0.6	0.6	1.0	0.6	1.0	0.6	0.5	0.6	0.5
of head (1/0)	(0.5)	(0.5)	(0.7)	(0.5)	(.)	(0.5)	(0.7)	(0.5)	(0.7)	(0.5)	(.)	(0.5)	(0.5)	(0.5)	(0.5)	(.)	(0.5)	(.)	(0.5)	(0.7)	(0.5)	(0.7)
Education of head	9.3	9.3	9.5	9.3	2.0	9.4	2.0***	9.4	2.0***	9.0	12.0	9.1	9.1	9.1	9.5	7.0	9.6	2.0	9.6	2.0***	9.6	2.0***
(years)	(3.4)	(3.4)	(3.5)	(3.3)	(.)	(3.3)	(2.8)	(3.3)	(2.8)	(3.2)	(.)	(3.2)	(3.2)	(3.2)	(3.6)	(.)	(3.5)	(.)	(3.4)	(2.8)	(3.4)	(2.8)
Number of adults	3.4	3.4	3.0	3.4	6.0	3.4	4.0	3.4	4.0	3.2	3.0	3.2	3.2	3.2	3.5	3.0	3.5	6.0	3.5	4.0	3.5	4.0
(count)	(1.5)	(1.5)	(0.0)	(1.5)	(.)	(1.5)	(1.4)	(1.5)	(1.4)	(1.5)	(.)	(1.4)	(1.4)	(1.4)	(1.5)	(.)	(1.5)	(.)	(1.5)	(1.4)	(1.5)	(1.4)
Number of children	2.5	2.5	2.0	2.5	3.0	2.5	2.0	2.5	2.0	2.5	2.0	2.4	2.4	2.4	2.6	2.0	2.6	3.0	2.6	2.0	2.6	2.0
(count)	(1.2)	(1.2)	(0.0)	(1.2)	(.)	(1.2)	(1.4)	(1.2)	(1.4)	(1.2)	(.)	(1.2)	(1.2)	(1.2)	(1.2)	(.)	(1.2)	(.)	(1.2)	(1.4)	(1.2)	(1.4)
Income per capita in	3469.0	3444.9	5638.2	3483.8	784.5	3486.5	1893.2	3486.5	1893.2	3521.0	6813.6	3558.0	3558.0	3558.0	3372.0	4462.8	3412.0	784.5	3416.5	1893.2	3416.5	1893.2
PPP $/year	(3863.3)	(3876.0)	(1662.3)	(3868.8)	(.)	(3877.3)	(2345.1)	(3877.3)	(2345.1)	(3979.7)	(.)	(3972.4)	(3972.4)	(3972.4)	(3794.6)	(.)	(3786.4)	(.)	(3802.6)	(2345.1)	(3802.6)	(2345.1)
Poverty rate (1/0)	0.2	0.2	0.0	0.2	0.0	0.1	0.5	0.1	0.5	0.2	0.0	0.2	0.2	0.2	0.1	0.0	0.1	0.0	0.1	0.5	0.1	0.5
	(0.4)	(0.4)	(0.0)	(0.4)	(.)	(0.4)	(0.7)	(0.4)	(0.7)	(0.4)	(.)	(0.4)	(0.4)	(0.4)	(0.3)	(.)	(0.3)	(.)	(0.3)	(0.7)	(0.3)	(0.7)
Farm size (acres)	1.3	1.3	1.9	1.3	2.2	1.3	1.9	1.3	1.9	1.3	0.5	1.3	1.3	1.3	1.2	3.3	1.2	2.2	1.2	1.9	1.2	1.9
	(1.2)	(1.2)	(1.9)	(1.2)	(.)	(1.2)	(1.5)	(1.2)	(1.5)	(1.2)	(.)	(1.2)	(1.2)	(1.2)	(1.1)	(.)	(1.1)	(.)	(1.1)	(1.5)	(1.1)	(1.5)

DB, double burden of malnutrition; DB 2, child is overweight (BAZ > +2 SD) and micronutrient-deficient; DB 2.1, child is overweight (WHZ > + 2SD) and micronutrient-deficient; DB 3, child overweight (BAZ > +2 SD) and stunting (HAZ < - 2 SD); DB 3.1, child overweight (WHZ > +2 SD) and stunting (HAZ < - 2 SD); n, sample size

Mean values are shown with standard deviations in parentheses. Differences in mean values are tested for significance: *p < .1, **p < .05, ***p < .01.
