# Supplementary material for: Various forms of double burden of malnutrition problems exist in rural Kenya
Source: BMC Public Health. 2019 Nov 21;19:1543. doi: 10.1186/s12889-019-7882-y (PMC6873738; doi:10.1186/s12889-019-7882-y)
Supplement: Supplementary file 7 — Additional file 7: Table S7. Prevalence of household-level DB within different subsamples [file 12889_2019_7882_MOESM7_ESM.pdf]

Additional Material A7

Table A7 Prevalence of household-level DB within different subsamples

	DB 4		DB 5		DB 6		DB 7		DB 8	
	n	%	n	%	n	%	n	%	n	%
Household (n=173)	1	0.6	5	2.9	22	12.7	1	0.6	30	17.3
Households with two adults and child (n=72)	0	0	2	2.8	11	15.3	0	0	20	27.8
Male adult-child pair (n=81)	0	0	2	2.5	11	13.6	0	0	20	24.7
Female adult-child pair (n=164)	1	0.6	5	3.1	22	13.4	1	0.6	30	18.3

DB, double burden of malnutrition; n, sample size

All DB definitions (DB 4-8) include adult overweight/obesity (BMI ≥25.0) and child undernutrition but differ in terms of the child undernutrition indicators used. DB 4, child underweight (BAZ <-2 SD); DB 5, child underweight (WAZ <-2 SD); DB 6, child stunting (HAZ <-2 SD); DB 7, child wasting (WHZ <-2 SD); DB 8, child is micronutrient-deficient.
