# Supplementary material for: Various forms of double burden of malnutrition problems exist in rural Kenya
Source: BMC Public Health. 2019 Nov 21;19:1543. doi: 10.1186/s12889-019-7882-y (PMC6873738; doi:10.1186/s12889-019-7882-y)
Supplement: Supplementary file 8 — Additional file 8: Table S8. Characteristics of households with and without household-level DB [file 12889_2019_7882_MOESM8_ESM.pdf]

Additional Material A8

Table A8 Characteristics of households with and without household-level DB

	Total (n=173)	Without DB 4 (n=172)	With DB 4 (n=1)	Without DB 5 (n=168)	With DB 5 (n=5)	Without DB 6 (n=151)	With DB 6 (n=22)	Without DB 7 (n=172)	With DB 7 (n=1)	Without DB 8 (n=143)	With DB 8 (n=30)
Kisii county (1/0)	0.7 (0.5)	0.7 (0.5)	1.0 (.)	0.7 (0.5)	0.8 (0.4)	0.7 (0.5)	0.7 (0.5)	0.7 (0.5)	1.0 (.)	0.7 (0.5)	0.8* (0.4)
Male household head (1/0)	0.8 (0.4)	0.8 (0.4)	0.0 (.)	0.8 (0.4)	0.4*** (0.5)	0.8 (0.4)	0.8 (0.4)	0.8 (0.4)	0.0 (.)	0.8 (0.4)	0.8 (0.4)
Age of household head (years)	46.4 (11.9)	46.3 (11.8)	66.0 (.)	46.4 (11.8)	47.8 (14.1)	46.5 (11.7)	45.6 (13.1)	46.3 (11.8)	66.0 (.)	45.5 (11.7)	50.8** (11.6)
Age of child (months)	35.9 (12.4)	35.8 (12.3)	59.0 (.)	35.6 (12.3)	45.0* (16.2)	35.3 (12.2)	40.3* (13.3)	35.8 (12.3)	59.0 (.)	35.0 (11.7)	40.1** (15.0)
Child breastfed (1/0)	1.0 (0.2)	1.0 (0.2)	1.0 (.)	1.0 (0.2)	0.8** (0.4)	1.0 (0.2)	1.0 (0.2)	1.0 (0.2)	1.0 (.)	1.0 (0.2)	1.0 (0.0)
Farming occupation of head (1/0)	0.6 (0.5)	0.6 (0.5)	1.0 (.)	0.6 (0.5)	1.0* (0.0)	0.6 (0.5)	0.6 (0.5)	0.6 (0.5)	1.0 (.)	0.6 (0.5)	0.7 (0.4)
Education of head (years)	9.3 (3.4)	9.4 (3.3)	2.0 (.)	9.4 (3.4)	6.4** (2.5)	9.4 (3.4)	8.5 (3.5)	9.4 (3.3)	2.0 (.)	9.5 (3.3)	8.4* (3.7)
Number of adults (count)	3.3 (1.5)	3.3 (1.4)	6.0 (.)	3.3 (1.4)	4.6* (1.7)	3.3 (1.5)	3.6 (1.5)	3.3 (1.4)	6.0 (.)	3.3 (1.5)	3.6 (1.3)
Number of children (count)	2.5 (1.1)	2.5 (1.2)	3.0 (.)	2.5 (1.2)	2.6 (1.1)	2.4 (1.2)	2.9* (1.0)	2.5 (1.2)	3.0 (.)	2.6 (1.2)	2.0*** (1.0)
Income per capita in PPP $/year	3563.3 (3948.3)	3579.6 (3954.2)	784.5 (.)	3525.5 (3836.9)	4816.0 (7297.8)	3513.0 (3747.1)	3922.2 (5267.7)	3579.6 (3954.2)	784.5 (.)	3323.2 (3598.6)	4691.6* (5222.2)
Poverty rate (1/0)	0.2 (0.4)	0.2 (0.4)	0.0 (.)	0.2 (0.4)	0.2 (0.4)	0.2 (0.4)	0.2 (0.4)	0.2 (0.4)	0.0 (.)	0.2 (0.4)	0.1 (0.3)
Farm size (acres)	1.3 (1.2)	1.3 (1.2)	2.2 (.)	1.3 (1.2)	1.4 (0.8)	1.3 (1.2)	1.2 (1.0)	1.3 (1.2)	2.2 (.)	1.1 (0.9)	1.9*** (2.0)

DB, double burden of malnutrition; n, sample size

All DB definitions (DB 4-8) include adult overweight/obesity (BMI ≥25.0) and child undernutrition but differ in terms of the child undernutrition indicators used. DB 4, child underweight (BAZ <-2 SD); DB 5, child underweight (WAZ <-2 SD); DB 6, child stunting (HAZ <-2 SD); DB 7, child wasting (WHZ <-2 SD); DB 8, child is micronutrient-deficient.

Mean values are shown with standard deviations in parentheses. Differences in mean values are tested for significance: *p < .1, **p < .05, ***p < .01.
